# How “simple” methodological decisions affect interpretation of population structure based on reduced representation library DNA sequencing: A case study using the lake whitefish

**DOI:** 10.1371/journal.pone.0226608

**Published:** 2020-01-24

**Authors:** Carly F. Graham, Douglas R. Boreham, Richard G. Manzon, Wendylee Stott, Joanna Y. Wilson, Christopher M. Somers

**Affiliations:** 1 Department of Biology, University of Regina, Regina, Saskatchewan, Canada; 2 Medical Sciences, Northern Ontario School of Medicine, Greater Sudbury, Ontario, Canada; 3 Department of Fisheries and Wildlife, Michigan State University, East Lansing, Michigan, USA; 4 Department of Biology, McMaster University, Hamilton, Ontario, Canada; Clemson University, UNITED STATES

## Abstract

Reduced representation (RRL) sequencing approaches (e.g., RADSeq, genotyping by sequencing) require decisions about how much to invest in genome coverage and sequencing depth, as well as choices of values for adjustable bioinformatics parameters. To empirically explore the importance of these “simple” methodological decisions, we generated two independent sequencing libraries for the same 142 individual lake whitefish (*Coregonus clupeaformis*) using a nextRAD RRL approach: (1) a larger number of loci at low sequencing depth based on a 9mer (library A); and (2) fewer loci at higher sequencing depth based on a 10mer (library B). The fish were selected from populations with different levels of expected genetic subdivision. Each library was analyzed using the STACKS pipeline followed by three types of population structure assessment (F_ST_, DAPC and ADMIXTURE) with iterative increases in the stringency of sequencing depth and missing data requirements, as well as more specific *a priori* population maps. Library B was always able to resolve strong population differentiation in all three types of assessment regardless of the selected parameters, largely due to retention of more loci in analyses. In contrast, library A produced more variable results; increasing the minimum sequencing depth threshold (-m) resulted in a reduced number of retained loci, and therefore lost resolution at high -m values for F_ST_ and ADMIXTURE, but not DAPC. When detecting fine population differentiation, the population map influenced the number of loci and missing data, which generated artefacts in all downstream analyses tested. Similarly, when examining fine scale population subdivision, library B was robust to changing parameters but library A lost resolution depending on the parameter set. We used library B to examine actual subdivision in our study populations. All three types of analysis found complete subdivision among populations in Lake Huron, ON and Dore Lake, SK, Canada using 10,640 SNP loci. Weak population subdivision was detected in Lake Huron with fish from sites in the north-west, Search Bay, North Point and Hammond Bay, showing slight differentiation. Overall, we show that apparently simple decisions about library construction and bioinformatics parameters can have important impacts on the interpretation of population subdivision. Although potentially more costly on a per-locus basis, early investment in striking a balance between the number of loci and sequencing effort is well worth the reduced genomic coverage for population genetics studies. More conservative stringency settings on STACKS parameters lead to a final dataset that was more consistent and robust when examining both weak and strong population differentiation. Overall, we recommend that researchers approach “simple” methodological decisions with caution, especially when working on non-model species for the first time.

## Introduction

The field of molecular ecology has experienced a recent increase in the use of reduced representation library (RRL) sequencing approaches for population studies. This popularity was enabled by low cost sequencing platforms and efficient RRL approaches for non-model species, which allows for the sequencing of a targeted portion of the genome across a large number of individuals (reviewed by [1] and [2]; [3]). Several variations of the RRL approach exist, most using either restriction enzymes or probes to target homologous regions of the genome across individuals and populations. Popular techniques include restriction site associated DNA sequencing (RADSeq; [4,5]), double digest RADSeq (ddRADSeq; [6]), genotyping by sequencing (GBS; [7]), 2bRAD [8], 3RAD [9], Rapture [10], RADcap [11], and Nextera-tagmented reductively-amplified DNA sequencing (nextRAD; [12]). Each of these approaches enable simultaneous sequencing and genotyping of thousands of single nucleotide polymorphism (SNP) markers, and they have been used in a wide range of studies including population structure [13–15], local adaptation and selection [16–20] and phylogenomics [21,22]. RRL techniques and downstream bioinformatics analyses are evolving rapidly, and consistent guidance when making fairly “simple” methodological decisions on basic elements of study design may often not be available in the published literature.

Molecular ecologists using RRL approaches are faced with decisions ranging from sequencing effort to adjustable bioinformatics parameters that can profoundly impact the quality of SNP data, and thereby the strength of inference in population studies. Targeted genome coverage and sequencing depth have a major influence on the cost and the quality of the data generated [23]. Sequencing a higher proportion of the genome (coverage) provides more data per individual, and higher sequencing depth increases confidence in base calls and the ability to identify low frequency variants [24–28]. However, limited research funds force most researchers to seek a trade-off between sequencing effort and resolution power. In addition, following the generation of sequence data, methods for filtering and the identification of variable SNP sites can profoundly impact the number and quality of loci retained [26,29], and potentially the power of downstream analyses. Clear explorations of how these methodological decisions affect the quality and quantity of SNPs, as well as the interpretation of population structure, are required.

All bioinformatics programs have adjustable parameters influencing the stringency of requirements for SNP calls that can influence the quantity and quality of loci in the final dataset. There are several programs designed to perform all steps in de novo SNP detection and genotyping from RRL data including STACKS [30], UNEAK [31] and pyRAD [32]. In this study, we chose STACKS because it is one of the most widely used programs for species without reference genomes, and there is previous research investigating error rates [26], parameter optimization [26,29,33,34] and influences on the number of markers in the final dataset [29] using this pipeline. In STACKS, SNP locus discovery and genotyping is done using three modules, *ustacks*, *cstacks* and *sstacks* [30,34]. The *ustacks* module first assembles stacks based on similarity on an individual level and *cstacks* then merges individual stacks into a population level catalog [34]. The *sstacks* module is then used to match individual stacks back to the catalog and the *populations* script outputs the data into various different formats [30]. In *ustacks* and *cstacks* there are three main parameters that control catalog construction: (1) minimum sequencing depth to create stacks (-m); (2) maximum number of mismatches allowed between alleles (-M); and (3) number of mismatches allowed between stacks (-n; [30,33,34]). In the *populations* module there are also parameters that affect the output and potential downstream analyses, including: (1) population map used (-M); (2) minimum percentage of individuals required to have the locus genotyped (-r); and (3) the number of populations required to have the locus genotyped (-p; [30,33,34]). These parameters can drastically influence the SNP dataset that is generated, but the impacts on downstream analyses and inferences about population subdivision have not been fully investigated.

Previous research has investigated how the parameters in the different STACKS modules influence SNP datasets generated, but few have examined how these changes actually influence population differentiation analyses. Paris et al. [33] developed the r80 rule, based on the generation of polymorphic loci across 80% of the population, to optimize important STACKS parameters (-m, -M and -n), as these may vary by study organism. Increasing -m and -M values generates fewer loci in the final dataset as a result of removal of loci lacking minimum sequencing depth, and over-merging of loci, respectively [26,29]. Studies examining the impact of sample size, study design, individual sequencing depth, and sequence quality have shown that fewer individuals (3–5 per site) and moderate sequencing depth (~10X) are often adequate for the detection of population subdivision and group assignment, even though higher sequencing depths result in more accurate genotype calls [35–38]. However, many of these studies are simulations and there is currently no consensus on sequencing depth requirements for population studies of non-model organisms, such as fish and wildlife.

RRL sequencing often results in missing data (loci, genotypes) in individuals and populations [39]; this issue may confound population structure analyses and it can be exacerbated by the selection of bioinformatics parameters. Multiple sources can give rise to missing data including issues during library preparation, such as size selection, DNA quality and uneven amplification, and biological sources, such as mutations within restriction or primer binding sites [39]. Mutations within binding sites, allelic drop out, can result in biased summary statistics and increased error rates [3,40–45]. This increase in allelic drop out has shown to negatively impact linkage mapping studies [46] but does not have significant impacts in RADSeq studies [3,42]. Shafer et al. [47] showed that increasing missing thresholds in the STACKS pipeline did not affect the summary statistics generated, such as heterozygosity, inbreeding coefficient, and the transition to transversion ratio. However, missing data becomes a more prominent issue at low sequencing depths, and previous research has shown that high-stringency filters can result in too few loci to discern population subdivision [48,49]. Further, Huang & Knowles [50] found that high stringency filters on missing data thresholds reduced the amount of missing data but may bias the resulting dataset by limiting the mutation spectrum included in downstream analyses. Missing data has also been investigated in phylogenetic studies using RRL datasets, in which less stringent thresholds on missing data resulted in larger datasets, increasing resolution in the phylogenetic analyses [21,39,50–54]. The interaction between library quality (genomic coverage and sequencing depth), bioinformatics parameter selection, and missing data, and their potential influences on the interpretation of population structure analyses require additional investigation. It is especially important for groups switching from more traditional markers (e.g., microsatellite DNA) to RRLs and SNPs to have access to such information to guide study design.

Here, we examine the influence of two different sequencing libraries and adjustment of various bioinformatics parameters on the resolution of strong and weak population subdivision in lake whitefish (*Coregonus clupeaformis*). Our overall objective was to provide an empirical example that will help molecular ecologists understand important influences on library and dataset quality, and aid in decision making about bioinformatics parameters. We generated nextRAD SNP data for 142 lake whitefish individuals from two different lakes: Lake Huron, Ontario, and Dore Lake, Saskatchewan, Canada. We chose nextRAD sequencing to accommodate both low quantity of input DNA and moderate levels of degradation as a result of field sampling conditions. We chose the lake whitefish as our study species because it is one of the most commercially harvested freshwater fish in the country, and it has been studied extensively in both population and evolutionary genetics contexts [55–61]. In addition, lake whitefish are an important component of food webs, facilitating transfer of energy from benthic to pelagic sources [62–65]. The lake whitefish is also part of a large-scale research program by our group investigating the influence of thermal effluents from nuclear power generation on development and population structure [9,66,67]. The analysis between the two lakes represents strong population subdivision, as indicated by different mitochondrial haplotypes resulting in distinct designatable units [60], while multiple sample sites within Lake Huron represent potential weak population subdivision. Our specific objectives were to: (1) examine how the trade-off between genomic coverage and sequencing depth influences the number of polymorphic loci, missing data, and other quality metrics; and (2) investigate how various bioinformatics parameters affect our interpretation of population subdivision.

## Materials and methods

### Study design

**Fig 1:**
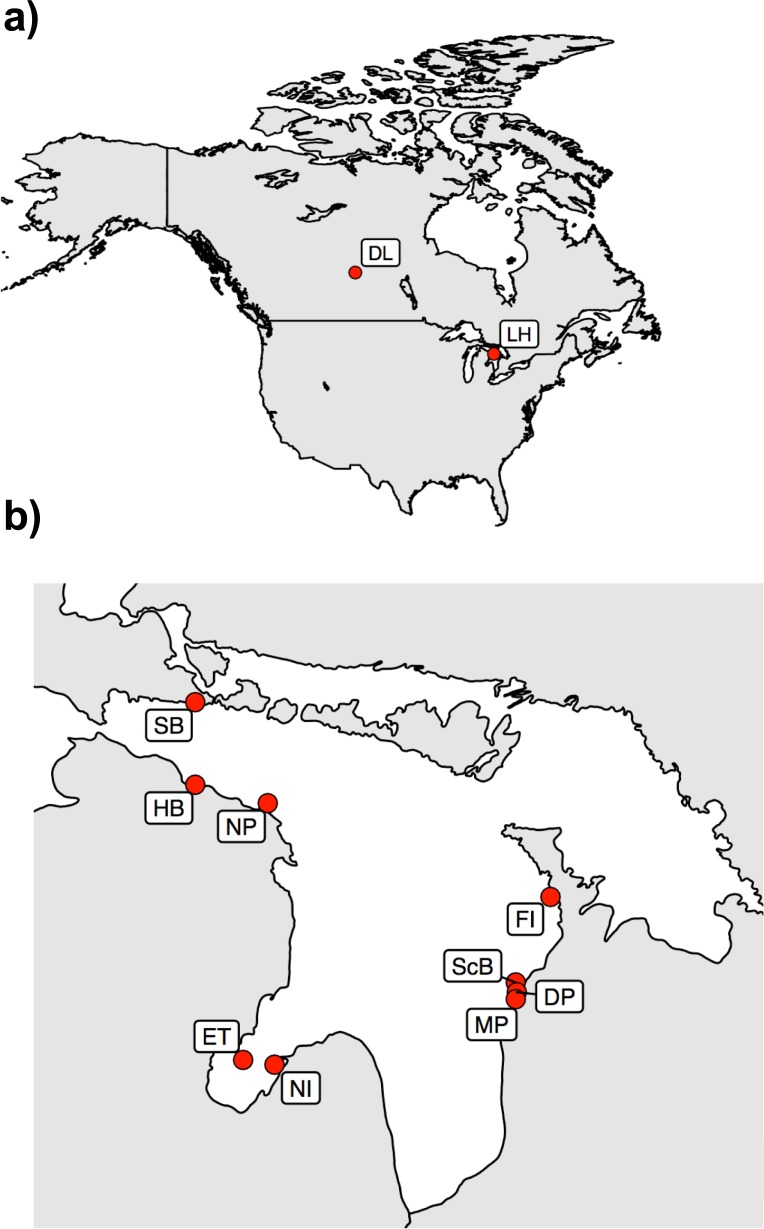
**Map of the two lakes sampled (a) and the nine sample sites in Lake Huron (b).** Fish were collected from Lake Huron sites in 2012 and Dore Lake in 2015. Site abbreviations can be found in Table 1.

We chose to perform an empirical examination of two different sequencing libraries and adjustable bioinformatics parameters by generating independent data using actual populations of our study species rather than performing simulations. This approach allowed us to focus on data generated from “real world” experiences rather than idealized or simulated data. We collected lake whitefish from 9 sites in Lake Huron, Ontario, Canada (44°48’N 82°24’ W), and Dore Lake, Saskatchewan, Canada (54°46’N 107°18’W; Fig 1). Fish from both of these lakes are descendants from the Mississippian refugium during the most recent glaciation event but have not been able to interbreed for at least several thousand years (strong population subdivision; [60]). In contrast, lake whitefish in different areas of Lake Huron face no physical barriers to dispersal and have previously shown only weak within-lake population subdivision using microsatellite data [68–71]. We designed our study to generate: (a) a library with higher genomic coverage (more loci) and lower sequencing depth based on a 9mer primer (library A); and (b) a library with lower coverage (fewer loci) but moderate sequencing depth based on a 10mer primer (library B). We then went through filtering and bioinformatics analyses iteratively, increasing the stringency of parameters, and compared the performance of datasets generated from the two libraries side-by-side in discerning whitefish population subdivision. We expected the signal of the between-lake population subdivision to be persistent across all analyses and used this as a benchmark for understanding when technical or analytical decisions had major impacts on the outcome. In contrast, we expected fluctuations in the signal from weak population subdivision within Lake Huron based on the resolving power of each dataset.

### Sample collection and DNA isolation

Adult spawning-phase lake whitefish were sampled from one site in Dore Lake, Saskatchewan and nine sites in Lake Huron, Ontario via commercial fishermen, fish processing plants and various management agencies (Table 1). Fish were terminally sampled using gillnetting and a 5 x 1 cm piece of dorsal muscle tissue was collected from 142 individuals and immediately frozen. A 20-mg subsample from each individual was stored in lysis buffer (4.0M urea / 0.2M NaCl / 0.1M Tris–HCl, pH 8.0 / 0.5% n-laurylsarcosine / 0.1 M 1,2-cyclo- hexanediamine) for genetic analyses. More detail on sampling can be found in Eberts et al. [66]. All animal research was approved by the University of Regina President's Committee on Animal Care, following the guidelines of the Canadian Council on Animal Care. The approved Animal Use Protocol was AUP 11–13 "Population and Conservation Genetics of Freshwater Fish".

**Table 1:** Collection data for 142 lake whitefish (*Coregonus clupeaformis*) samples from Lake Huron, ON, Canada, and Dore Lake, SK, Canada.

Site	Lake Location	Latitude	Longitude	Collection Date	Total (n)
Lake Huron					
ET	East Tawas	43.906	-83.672	Nov 13, 2012	14
NI	North Island	43.878	-83.435	Nov 15, 2012	14
NP	North Point	45.395	-83.486	Nov 1, 2012	14
HB	Hammond Bay	45.502	-84.033	Nov 4, 2012	14
SB	Search Bay	45.981	-84.497	Nov 2, 2012	15
ScB	Scougall Bank	44.355	-81.617	Nov 6–18, 2012	17
DP	Douglas Point	41.298	-81.609	Nov 6–15, 2012	9
MP	McRae Point	44.258	-81.617	Nov 7–18, 2012	17
FI	Fishing Islands	44.709	-80.312	Nov 18, 2012	14
Dore Lake					
DL	Dore Lake	54.767	-107.300	Nov 22, 2015	14

Genomic DNA was isolated from 20 mg of dorsal muscle tissue following manufacturers guidelines (Genomic DNA Isolation Kit, Norgen Biotech Corp., Ontario, Canada) except for extending the proteinase K digestion to 8–12 hours and the addition of 28 U of RNAse A (Qiagen Inc., Ontario, Canada). DNA was quantified using a Qubit 2.0 Fluorometer (Life Technologies Inc., Ontario, Canada) and DNA quality (level of shearing) was assessed using an E-Gel (Thermo Fisher Scientific, Canada).

### NextRAD sequencing

We used an amplification-based RRL approach to accommodate variation in DNA quality among individuals and low amounts of input DNA for library preparation. Each sample was sequenced independently in each library with identical input DNA. Genomic DNA was converted into nextRAD genotyping-by-sequencing libraries (SNPsaurus, Oregon, USA) as described by Russello et al. [12]. Briefly, genomic DNA was first digested with the Nextera reagent (Illumina, Inc., British Columbia, Canada), which randomly fragments the genome using a transposase. The Nextera reagent also ligates short adapter sequences to the ends of the fragments. For high quality (mostly intact, high molecular weight DNA) samples the Nextera reaction included 20 ng of input DNA; for moderately degraded (sheared; fragments < 5 Kb) samples we used 40–60 ng of input DNA to compensate for degradation. Fragmented DNA was then amplified with a primer matching the adaptor sequence and extending either 9 (library A) or 10 (library B) nucleotides into the genomic DNA with the selective sequences 5’-GTGTAGAGC-3’ and 5’-GTGTAGAGCC-3’, respectively. These two primers were used to create two completely independent libraries with different selectivity. Following hybridization of the primers, PCR amplification was done with an annealing temperature of 72°C for 27 cycles. This allowed for selective hybridization and amplification of fragments that paired with the primer sequence as well as the incorporation of individual barcodes. The nextRAD libraries were then sequenced on an Illumina HiSeq 4000 on a total of six lanes using single-end 150 bp reads (University of Oregon, Oregon, USA).

### Data analysis

#### Data quality filtering

**Fig 2:**
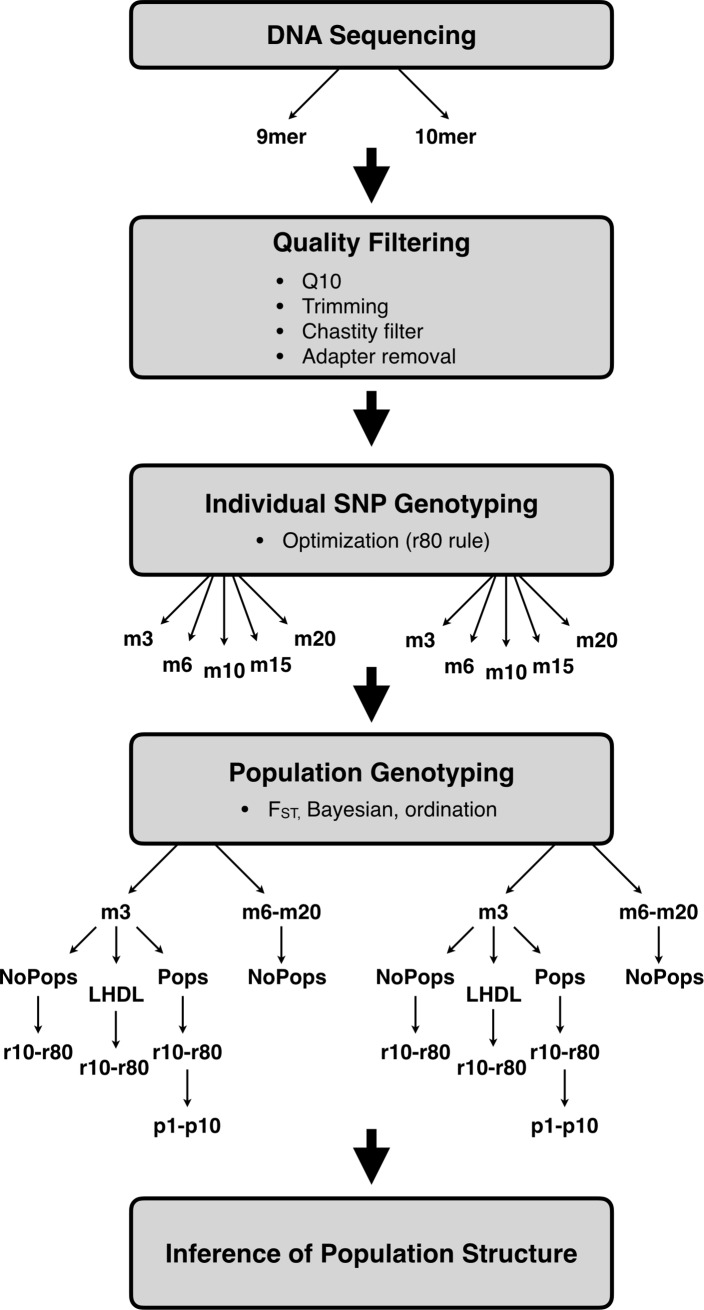
Flow chart of the bioinformatics pipeline and decisions required for analysis. Each arrow indicates different libraries that were generated in the analysis.

FASTQ files were first processed using *Trimmomatic* [72] to remove the Nextera adaptors (Fig 2). The remaining reads were then visualized in FastQC to ensure effective adaptor removal [73]. All sequences were analyzed using STACKS 2.0 beta 7 [30,74]. Further quality filtering was done using *process_radtags* to remove any reads with uncalled bases, discard reads with an average quality score below Q10 or failed the Illumina chastity filter and truncate the reads to 150 bps (Fig 2).

#### SNP genotyping parameters

Following quality filtering, the maximum distance allowed between stacks (-M in *ustacks*) and the number of mismatches allowed between sample loci when building the catalog (-n in *cstacks*) were optimized as recommended by Paris et al. ([33]; Fig 2). The maximum distance between stacks (-M) parameter was tested from 1–4 as a result of the highly repetitive nature of the lake whitefish genome. The number of mismatches allowed between samples when building the catalog was then tested from 0–2. Following parameter optimization, the *denovo_map*.*pl* script was used to run the STACKS pipeline. This script was run five times on each sequencing library, A and B, with the minimum sequencing depth (-m) set at 3, 6, 10, 15 and 20 (Fig 2). By filtering for read depth at the *ustacks* level instead of the *populations* level it allows for better performance of the SNP model, which identifies variable sites in the *ustacks* module [33]. While the -m parameters varied in each iteration, the other parameters were held constant. In *ustacks*, a maximum distance between stacks of 1 (-M) was used and the varying–m value as stated above. The removal algorithm was enabled to eliminate highly-repetitive stacks, which should limit the number of highly repetitive loci included. The catalog was generated in *cstacks* using a mismatch value between samples (-n) of 1 as determined above. Finally, individual stacks were then searched against the catalog using *sstacks*.

#### Population genotyping parameters

For each parameter set (m3 –m20), the *populations* script was used to export SNPs with a minor allele frequency greater than 0.05. In both sequencing libraries, a range of the proportion of individuals within the population containing the locus (-r), the minimum number of defined populations with the locus with sample site designation (-p), and different population maps (-M) were tested in the *populations* script (Fig 2). Each of these different values was varied in turn while the other values remained constant. The minimum percentage of individuals in a population required to process a locus (-r) was tested from 0.1–0.8 (Fig 2). These -r values were tested with three different population maps: (1) no specified populations (NoPops), (2) Lake Huron and Dore Lake populations (LHDL), and (3) sample site designations (Pops; Fig 2). While varying the -r flag in the script, the -p value was held constant at 1, indicating that the locus only needed to be present at the specified percentage in 1 population. Different -p values were also tested using the Pops population map and r30 on the m3 sequencing parameter dataset. The -p value was tested from p1 –p10, increasing the number of defined populations that were required to contain the loci in greater than 30% of the individuals (Fig 2). When comparing the different population maps, a value of p1 was used in order to compare different levels of loci and missing data across the datasets. All three of these parameters, -p, -r and -popmap, influence the level of missing data included in the final matrix. By changing the stringency on these parameters iteratively we were able to examine how missing data influences the final data matrix and the inference of population subdivision.

The level of missing genotypes per individual was examined using *grur* [75]. A principal coordinate analysis was run based on the presence/absence of genotypes within the samples to create an isolation by missingness (IBM) plot. This IBM plot can be used to determine if samples are related to each other based on the missing genotypes in the data. Loci were then checked for conformation to Hardy Weinberg Equilibrium (HWE; P < 0.05) using PLINK 1.07 [76]. Loci that did not conform to HWE in both the Lake Huron and Dore Lake populations were used to create a blacklist and were omitted from future analyses.

### Population differentiation

After filtering, we performed population structure analyses on the SNP data generated from each parameter set using pairwise fixation indices (F_ST_; GENODIVE; [77,78]), ordination (DAPC; [79]), and maximum likelihood approaches (ADMIXTURE; [80,81]; Fig 2). The population differentiation values from each analysis were used to compare the outcomes across the different datasets generated from the modifications listed above. As a result of assumptions from each analysis, both the pairwise fixation indices (F_ST_) and maximum likelihood analyses were conducted using only the loci in HWE, while the ordination analysis was conducted with all loci. We calculated pairwise fixation indices [77] using the program GENODIVE with 5,000 permutations. F_ST_ values were evaluated by comparing individual sampling sites. The program ADMIXTURE was then used to estimate ancestry using a maximum likelihood approach. ADMIXTURE uses a block relaxation approach coupled with novel Quasi-Newton acceleration of convergence [80]. In order to determine the correct number of distinct populations (K), the ADMIXTURE program uses a cross-validation approach. The R package *pophelper* [82] was used to visualize the K = 2 and K = 3 data from ADMIXTURE. Finally, the data were analysed using discriminant analysis of principle components (DAPC), a multivariate ordination approach, implemented in *adegenet* [79]. The DAPC plot was generated using N/3 principle components to avoid over fitting the data and for consistency across all analyses. All analyses were conducted on a regional super computer (Breezy, Westgrid, Compute Canada, Canada).

## Results

### Data analysis

#### Data quality filtering

The steps to generate libraries A and B were identical, with the exception of using a different primer to capture loci for sequencing (9mer vs. 10mer). Correspondingly, the total number of reads generated in each library was very similar, at 296,073,514 and 297,243,177 for library A and B, respectively. The total number of reads per individual following the removal of the Nextera adapters was also similar at 2,070,281 (SD = 390,050) and 2,078,327 (SD = 742,956) in library A and B, respectively. However, following *process_radtags*, library A had an average of 1,311,336 (SD = 356,212) and library B had an average of 1,875,843 (SD = 739,513) reads per individual remaining. The difference between the two datasets resulted from library A losing an average of 758,945 (SD = 114,274) reads per individual due to low read quality scores during filtering in *process_radtags*, whereas library B only lost an average of only 203,279 reads (SD = 25,838). The difference in library quality was due to the 9mer probe hybridizing with many more highly similar regions in the whitefish genome than the 10mer, which caused a nucleotide diversity issue on the Illumina sequencer. PhiX DNA in identical quantities was used to offset this diversity issue in both libraries, but library A was more negatively affected.

#### SNP genotyping parameters

**Fig 3:**
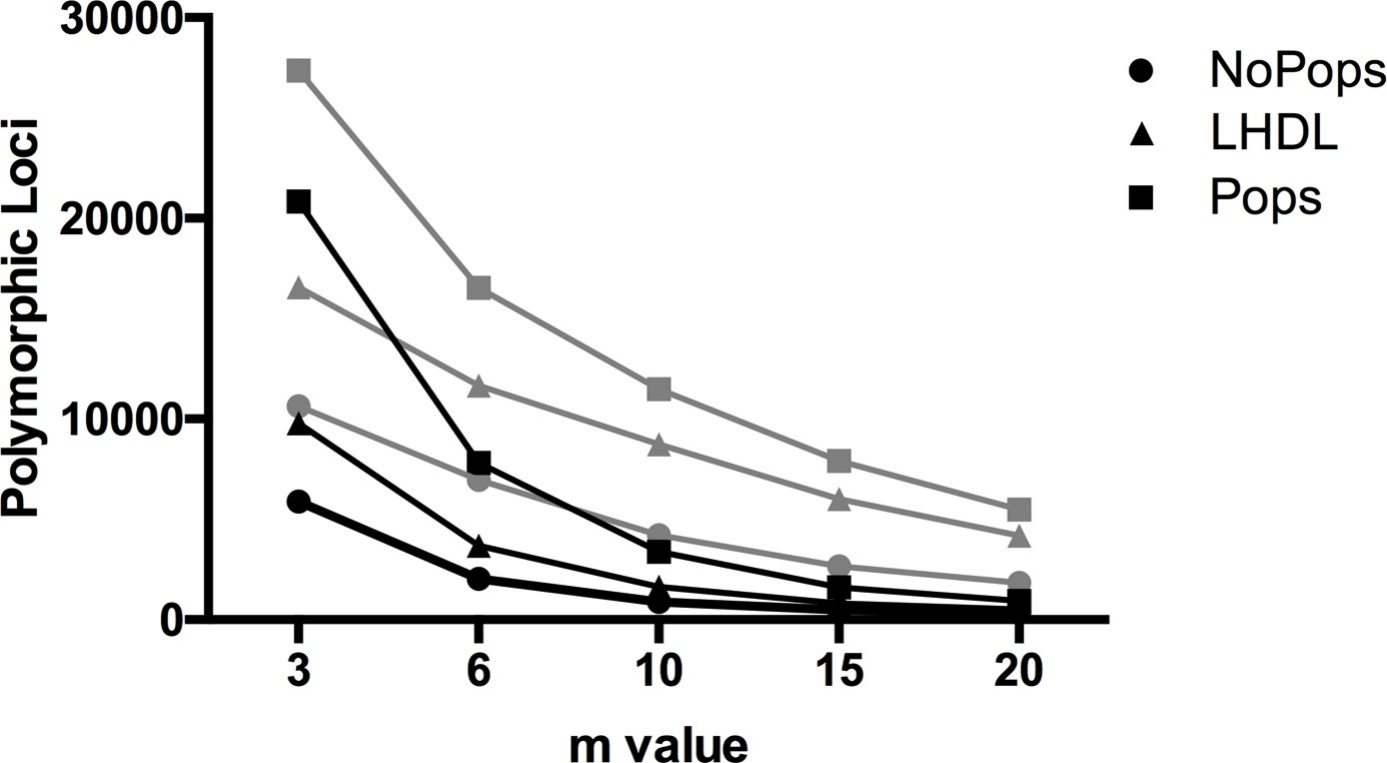
**Total number of polymorphic loci generated with five different levels of sequencing depth (-m) in *ustacks* in library A (black) and B (grey).** Data were generated using three different population maps in the *populations* module, no specified populations (NoPops), Lake Huron and Dore Lake specifications (LHDL) and sample sites (Pops). Library A was generated using a 9mer probe and library B was generated using a 10mer probe.

The r80 rule, the increment with the higher level of polymorphic loci present in 80% of the populations, was used to determine that M = 1 (one substitution per stack in *ustacks*) and n = 1 (one mismatch allowed between loci within the catalog in *cstacks*) were optimal for the lake whitefish genome and were used for all following analyses [33]. Library A and B were filtered with five different sequencing depth (-m) cut-offs, m3, m6, m10, m15 and m20, in the *ustacks* module of *denovo_map*.*pl*. As expected, increasing the stringency of the -m criterion drastically increased the average sequencing depth per locus and decreased the total number of matched loci in each individual for both library A and B (Table 2). Even at the m3 threshold, library A generated fewer loci per individual as a result of only 28.3% (SD = 7.4%) of the reads passing the threshold criteria compared to 58.2% (SD = 4.0%) in library B. This trend was consistent across all -m values and was likely the result of high levels of repeats as indicated by the number of blacklisted stacks in *ustacks*, with an average of 8071 (SD = 1501) per individual in library A and 4537 (SD = 1498) per individual in library B. These results indicate that the reduced number of loci in library A resulted from both a drop in read quality and fewer loci passing the sequencing depth threshold in *ustacks*. Library B consistently had more than 2X as many loci in the catalog and matched loci following the *cstacks* and *sstacks* modules regardless of the -m value used (Table 2). Following the *populations* module, the total number of polymorphic loci varied depending on the specified sequencing depth in the *ustacks* module (Table 2). Library B had 1.8–5.4X more polymorphic loci than library A across all -m values, with the largest loss of polymorphic loci occurring at m6 in the library A (Fig 3; Table 2).

**Table 2:** Summary data from each sequencing library, A and B, with increasing sequencing depth (-m) thresholds in the *ustacks* module of STACKS. The m parameter influences the number of loci generated per individual in the *ustacks* module of STACKS, which influences the total number of loci in the catalog and the matched loci in the *sstacks* module. The *populations* module was then run on each library independently using no population differentiation in the population map (NoPops), one population required to contain the locus (-p) and 30% of the individuals required to contain the locus (-r). Library A was generated using a 9mer probe, while library B was generated using a 10mer probe.

**Library A**					
	m3	m6	m10	m15	m20
Average depth (per individual)	9.01 (SD = 1.0935)	21.09 (SD = 2.7930)	44.57 (SD = 9.6451)	82.99 (SD = 19.9276)	127.78 (SD = 28.7960)
Total Loci in catalog	1,381,311	467,790	185,070	84,677	47,509
Matched loci (per individual)	39,112 (SD = 14,653.63)	13,600 (SD = 9,286.50)	5,594 (SD = 5,358.70)	2,677 (SD = 3,045.56)	1,603 (SD = 1,892.85)
Loci post *populations* (p1r30NoPops)	8,606	2,669	1,193	724	555
Polymorphic loci	5,882	2,029	884	479	337
HWE <0.05	5,613	1,859	744	369	245
**Library B**					
Average depth (per individual)	12.24 (SD = 2.7465)	20.73 (SD = 2.8198)	31.87 (SD = 2.3186)	46.54 (SD = 2.1598)	62.03 (SD = 3.2270)
Total Loci in catalog	3,742,031	1,874,424	987,836	538,722	329,949
Matched loci (per individual)	84,912 (SD = 21,703.38)	44,473 (SD = 17,336.98)	25,345 (SD = 12,639.20)	15,205 (SD = 8,853.66)	10,179 (SD = 6,423.01)
Loci post *populations* (p1r30NoPops)	18,857	12,398	8,132	5,484	3,972
Polymorphic loci	10,640	6,955	4,202	2,669	1,834
HWE <0.05	9,942	6,518	3,897	2,438	1,641

**Fig 4:**
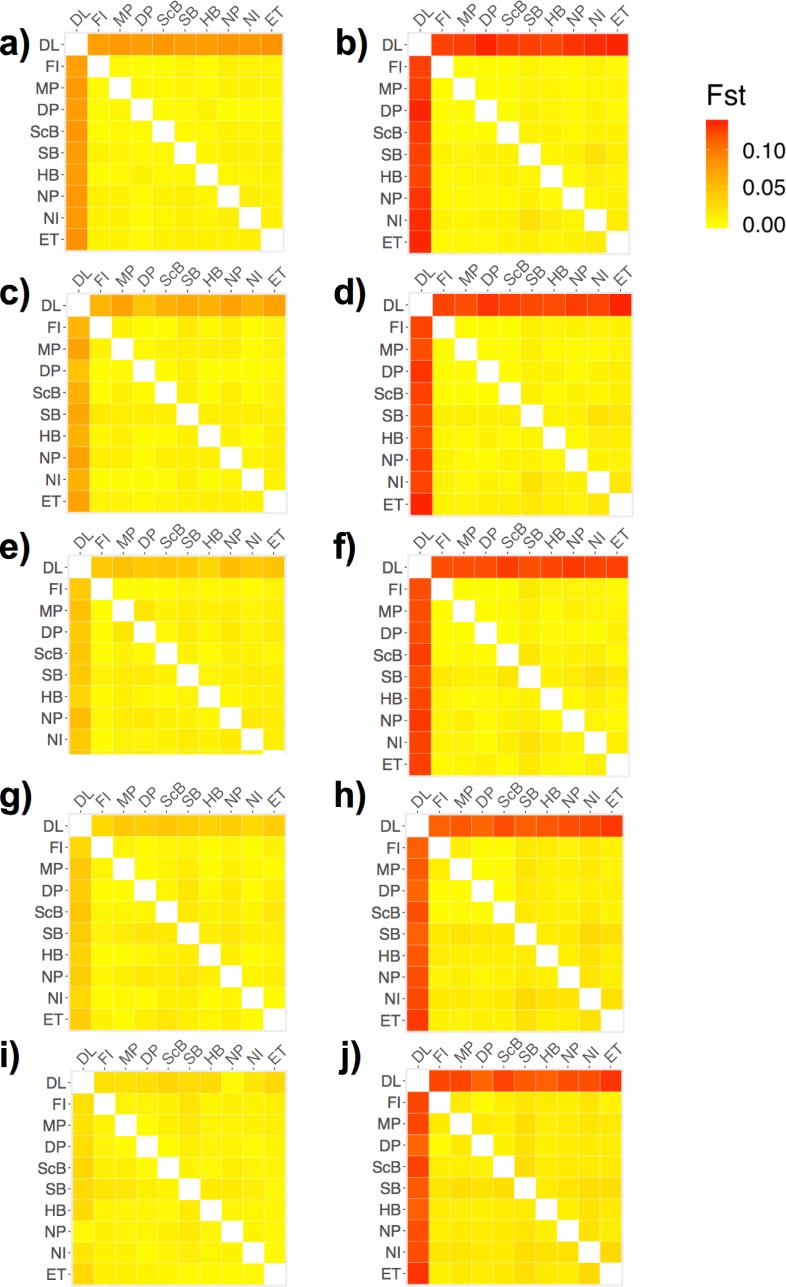
**Heatmap representing the pairwise fixation indices (F**_**ST**_**) estimated using GENODIVE across all sample sites with increasing sequencing depth (-m) in the *ustacks* module with m3 (a, b), m6 (c, d), m10 (e, f), m15 (g, h) and m20 (i, j) in library A (a, c, e, g, i) and B (b, d, f, h, j).** Larger F_ST_ values represent larger population differentiation. Site abbreviations can be found in Table 1.

**Fig 5:**
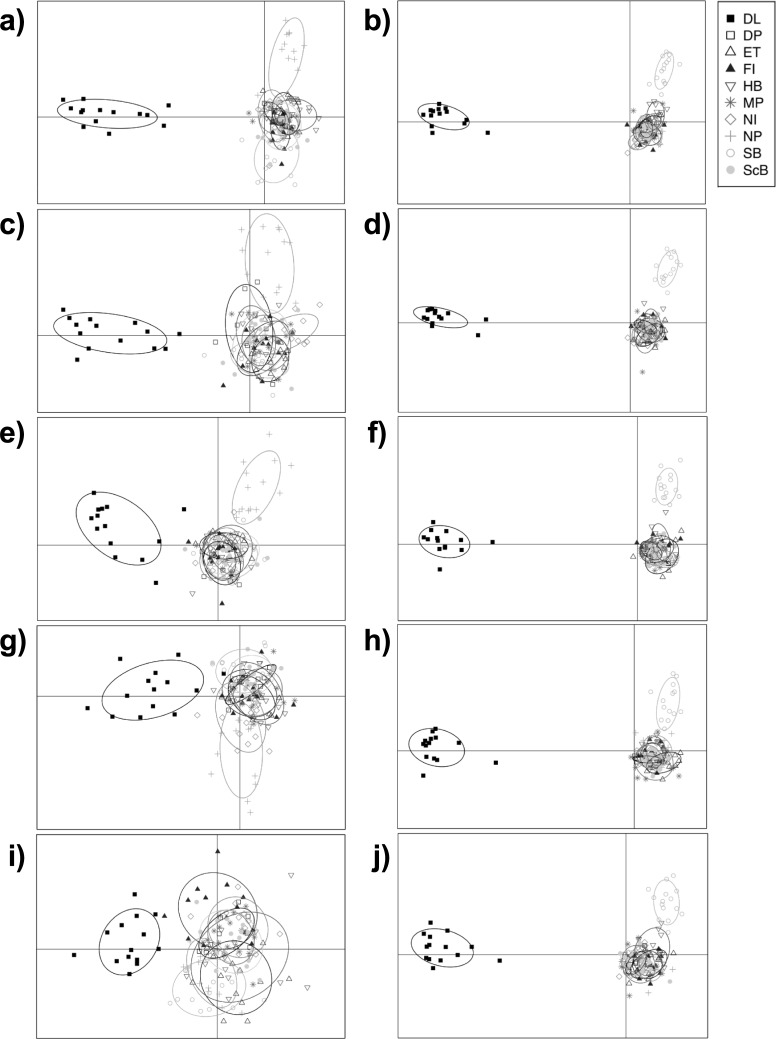
**Discriminant analysis of principle components (DAPC) of all sample sites with increasing sequencing depth (-m) in the *ustacks* module with m3 (a, b), m6 (c, d), m10 (e, f), m15 (g, h) and m20 (i, j) in library A (a, c, e, g, i) and B (b, d, f, h, j).** The DAPC analysis was run with 46 principle components (N/3) for the analysis. Distinct ellipses indicate population differentiation. Site abbreviations can be found in Table 1.

**Fig 6:**
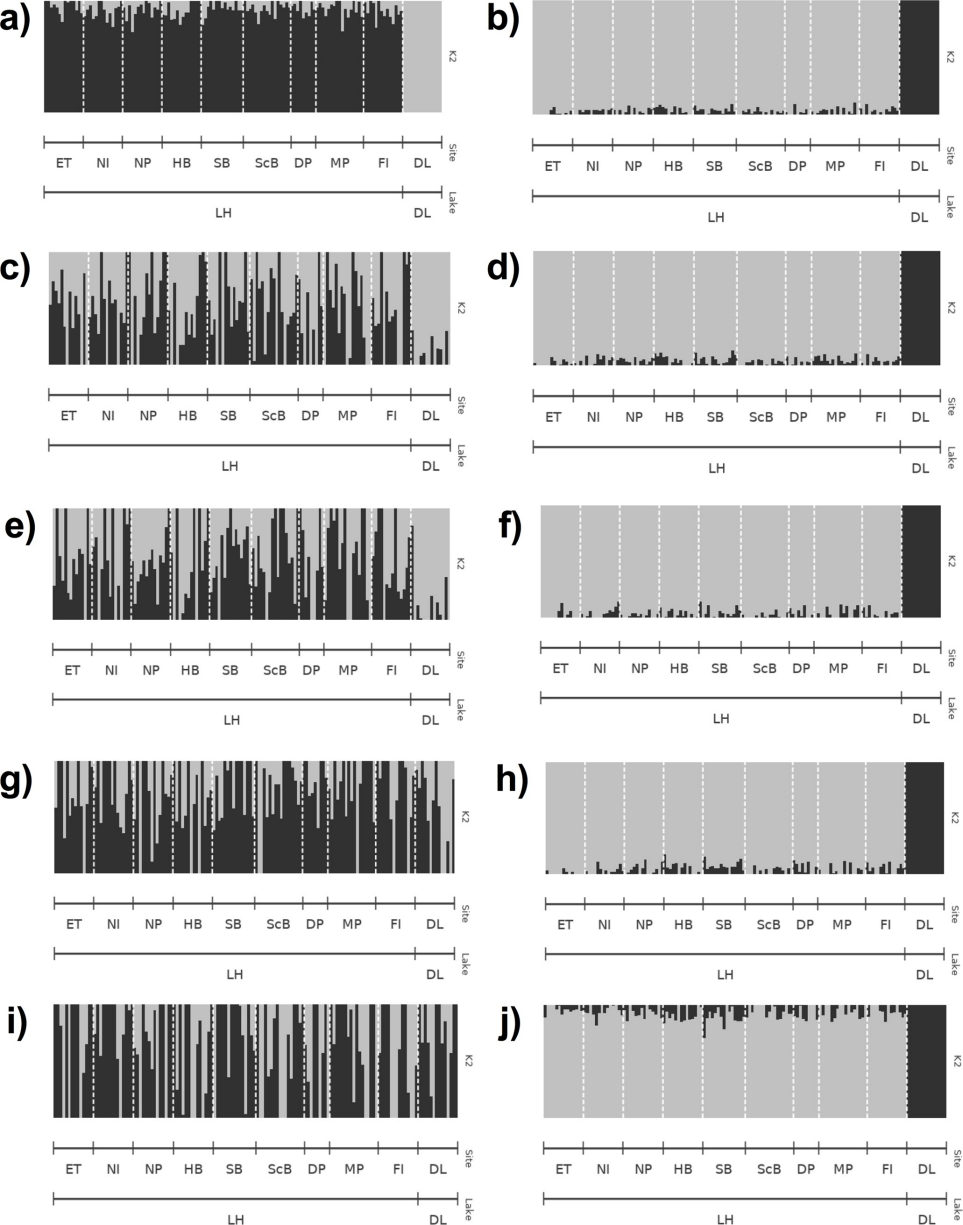
**ADMIXTURE analysis of all sample sites with increasing sequencing depth (-m) in the *ustacks* module with m3 (a, b), m6 (c, d), m10 (e, f), m15 (g, h) and m20 (i, j) in library A (a, c, e, g, i) and B (b, d, f, h, j).** K2 was used as it had the lowest cross-validation value post-hoc. Each line represents an individual from the corresponding sample site. Site abbreviations can be found in Table 1.

The resolution of strong (between-lake) population subdivision was affected by the different sequencing depth thresholds used to generate the dataset. Mean overall F_ST_ values between Dore Lake and each site in Lake Huron were low in library A and decreased with more stringent sequencing depth values (-m parameter; Fig 4A, 4C, 4E, 4G and 4I): m3 = 0.0776 (SD = 0.0031), m6 = 0.0536 (SD = 0.0078), m10 = 0.0413 (SD = 0.0054), m15 = 0.0337 (SD = 0.0054) and m20 = 0.01922 (SD = 0.0087). Some sites did not appear differentiated between the two lakes using the F_ST_ approach with high sequencing depth parameters (m20), as a result of the small number of loci remaining in library A (Fig 4I). Search Bay (SB) was consistently differentiated from the rest of the Lake Huron sites in all datasets and North Point (NP), East Tawas (ET) and North Island (NI) showed potential differentiation with increasing m values in library A (Fig 4A, 4C, 4E, 4G and 4I). Mean overall F_ST_ values were higher at all sequencing depth thresholds in library B (Fig 4B, 4D, 4F, 4H and 4J): m3 = 0.1311 (SD = 0.0038), m6 = 0.1289 (SD = 0.0041), m10 = 0.1282 (SD = 0.0034), m15 = 0.1241 (SD = 0.0069) and m20 = 0.1302 (SD = 0.0078), and all comparisons between Dore Lake and Lake Huron resulted in significant (P < 0.05) differentiation (data not shown). Library B and higher m values in library A showed differentiation of Search Bay (SB) from other Lake Huron Sites (S1 Fig). Sites found on the eastern shores, Scougall Bank (ScB), Douglas Point (DP), McRae Point (MP) and Fishing Islands (FI) also showed slight differentiation in library B (S1B, S1D, S1F, S1H and S1J Fig). DAPC clearly resolved population differentiation between Lake Huron and Dore Lake in both library A and B (Fig 5). Assignment proportions ranged from 0.7324 to 0.7606 in library A, and 0.7676 to 0.7887 in library B. Within Lake Huron, North Point (NP) and Search Bay (SB) showed weak differentiation in library A until the m15 dataset and North Island (NI) showed subdivision at m3 (S2A, S2C, S2E, S2G and S2I Fig). In library B, Search Bay (SB) and North Point (NP) both showed consistent genetic subdivision in all sequencing depth datasets (S2B, S2D, S2F, S2H and S2J Fig). ADMIXTURE clearly detected strong population subdivision at m3 in library A. Two groups representing Dore Lake and Lake Huron were still evident until m15, but with much less clarity (Fig 6A, 6C and 6E); however, no signal of subdivision between the lakes remained when the m20 value was applied. In contrast, library B was always able to resolve population differentiation between the two lakes at all sequencing depth thresholds with high average ancestry fraction values (Fig 6B, 6D and 6F). No differentiation between sites in Lake Huron was detected in library A, while North Point (NP), Hammond Bay (HB) and Search Bay (SB) are differentiated in all m datasets in library B (S3 Fig).

#### Population genotyping and differentiation parameters

**Fig 7:**
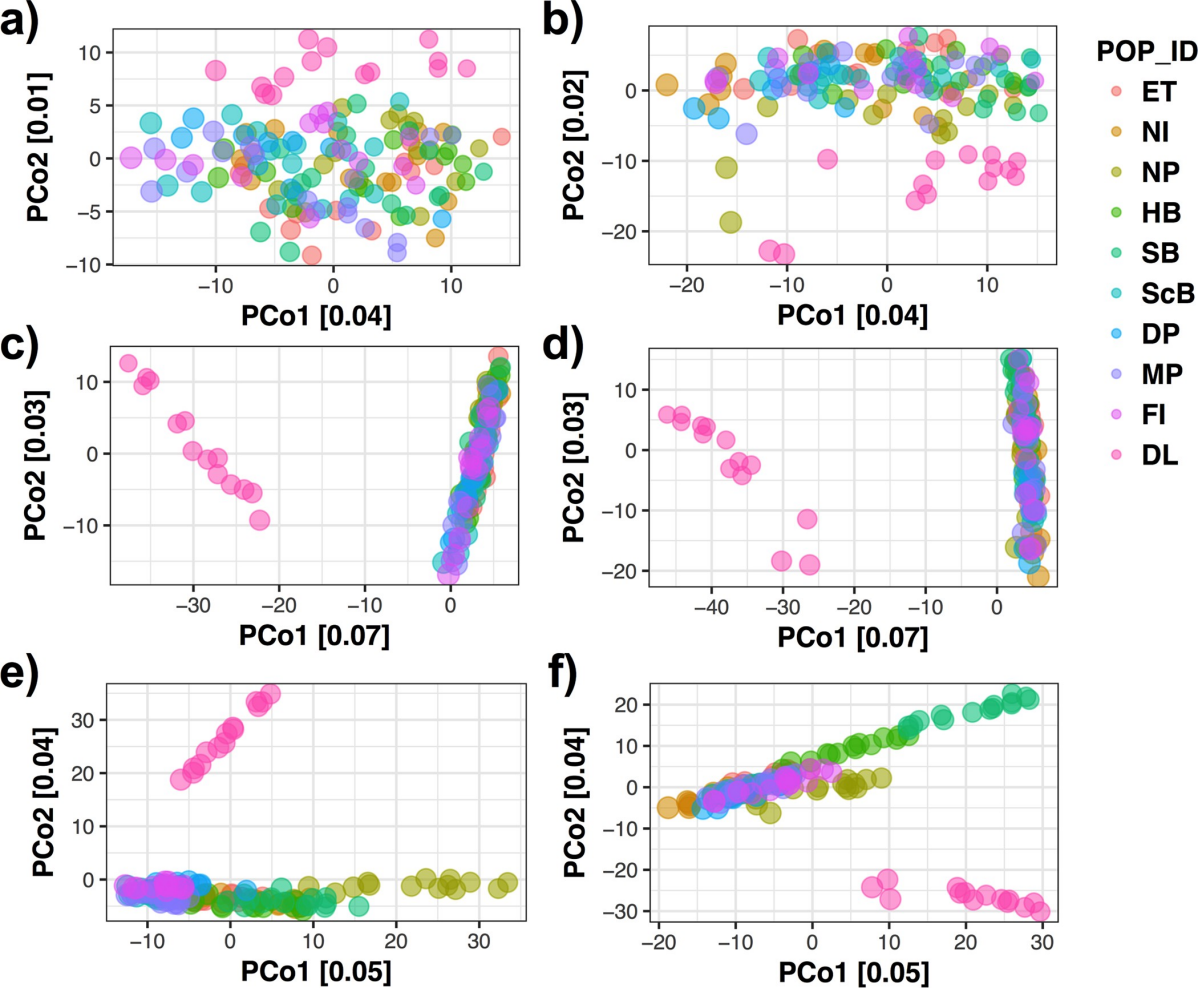
**Identity-by-missingness (IBM) plot generated using *grur* with 3 different population maps in the *population* module of STACKS: (1) no population designation (NoPops; a, b), (2) Lake Huron and Dore Lake designations (LHDL; c, d) and (3) sample site designations (Pops; e, f) in library A (a, c, e) and B (b, d, f).** Site abbreviations can be found in Table 1. The IBM analysis is conducted using a Principal Coordinates Analysis (PCoA) to determine clustering based on missing data. The x and y axes represent the first and second principal coordinate, respectively.

There were more polymorphic loci in library B in every population map and minimum sequencing depth (-m) permutation tested (Fig 3). Further, the number of polymorphic loci generated was always higher with the minimum sequencing depth (-m) in *ustacks* set at three, with ~20X and ~5X more loci in the m3 datasets compared to m20 in library A and B, respectively. More SNP loci were generated using the Pops population map from r10-80 compared to both the NoPops and LHDL population maps (Fig 3 and Table 3). The NoPops population map resulted in a higher genotyping rate compared to the LHDL and Pops maps in both library A and B when comparing -m values, with each locus being genotyped in approximately double the number of individuals (S4 Fig). The high genotyping rate resulted in much less missing data using the NoPops population map. Missing data can lead to clustering of individuals based on biases in the absence of data rather than true biological relationships. The IBM plot generated using *grur* showed that both the LHDL and Pops population maps generated datasets with biases from missing data (Fig 7). The NoPops population map resulted in IBM plots with slight Dore Lake differentiation, likely as a result of biological differences between the populations, while both the LHDL and Pops population maps generated skewed IBM plots with significant differentiation based on missing data (Fig 7). Further, although we expect some biological missing data between Lake Huron and Dore Lake, this is exacerbated using both the LHDL and Pops population maps, as a result of increased missing data (Fig 7). However, in general the divergence among sites based on missing data in the NoPops population map was small, and likely did not impact our final interpretations.

**Table 3:** Summary data from each sequencing library, A and B, with varying sequencing depth (-m), genotyped percentage (-r) and number of populations (-p) values and population maps. The m parameter influences the number of loci generated per individual in the *ustacks* module of STACKS, which influences the total number of loci in the catalog and the matched loci in the *sstacks* module. The *populations* module was then run on each library independently using either no population differentiation (NoPops), lake population differentiation (LHDL) or sample site differentiation (Pops) in the population map. Both the number of populations required to have the locus genotyped (p) and the percentage of individuals required to have the locus (-r) were also varied in the *populations* module. Library A was generated using a 9mer probe, while library B was generated using a 10mer probe.

m Value	Population Map	r Value	p Value	Loci	Missing (Proportion)	Loci	Missing (Proportion)
				Library A		Library B	
3	NoPops	10	1	25664	0.77	29810	0.69
		20		11276	0.65	15827	0.54
		30		5882	0.55	10640	0.44
		40		3027	0.45	7673	0.35
		50		1649	0.35	5756	0.29
		60		911	0.27	4078	0.22
		70		513	0.20	2789	0.16
		80		239	0.13	1781	0.11
	LHDL	10	1	34832	0.82	43268	0.78
		20		19420	0.78	26919	0.72
		30		9781	0.72	16554	0.63
		40		6650	0.73	13244	0.61
		50		4692	0.74	10999	0.60
		60		2268	0.66	7158	0.52
		70		1446	0.67	5512	0.54
		80		497	0.55	2664	0.38
	Pops	10	1	74515	0.90	94841	0.88
		20		43172	0.87	52450	0.83
		30		20829	0.84	27494	0.75
		40		14755	0.82	21311	0.72
		50		10324	0.82	16502	0.69
		60		5444	0.80	11893	0.67
		70		3308	0.78	8812	0.64
		80		1381	0.75	5750	0.64
	Pops	30	1	20829	0.84	27494	0.75
		30	2	13329	0.76	15415	0.61
		30	3	9658	0.70	11031	0.52
		30	4	7431	0.65	10970	0.49
		30	5	5915	0.60	5826	0.41
		30	6	4776	0.56	5864	0.38
		30	7	3793	0.51	5923	0.35
		30	8	2943	0.46	5949	0.33
		30	9	2201	0.41	5938	0.31
		30	10	1439	0.34	4973	0.27
6	NoPops	30	1	2029	0.53	6955	0.46
	LHDL	30	1	3696	0.73	11663	0.67
	Pops	30	1	7815	0.84	16542	0.75
10	NoPops	30	1	884	0.51	4202	0.46
	LHDL	30	1	1635	0.72	8725	0.73
	Pops	30	1	3379	0.83	11485	0.77
15	NoPops	30	1	479	0.48	2669	0.46
	LHDL	30	1	811	0.68	6007	0.75
	Pops	30	1	1613	0.81	7915	0.78
20	NoPops	30	1	337	0.48	1834	0.47
	LHDL	30	1	524	0.66	4196	0.75
	Pops	30	1	929	0.79	5489	0.79

The three population maps, NoPops, LHDL and Pops, were also analyzed using three population differentiation approaches to examine the effects of missing data and genotyping rate on downstream analyses. These analyses were done with a minimum depth threshold value of m3, minimum of 30% individuals containing the locus (-r) and a minimum of one population containing the locus (-p). F_ST_ and DAPC analyses were both able to consistently differentiate the Dore Lake samples from each of the Lake Huron sample sites regardless of the population map in both sequencing libraries (S5 and S6 Figs). Regardless of the population map used, library B resulted in average F_ST_ values ~2X larger for the Dore Lake samples than library A (S5 Fig). Search Bay (SB) showed potential differentiation in the LHDL and Pops population maps in library A and in all population maps in library B (S5 Fig). When run independently, Search Bay (SB) showed slight differentiation using the LHDL and Pops population maps in library A, while it was differentiated using all population maps in library B (S7 Fig). Library B also showed differentiation of sites in eastern Lake Huron, Fishing Islands (FI), McRae Point (MP), Douglas Point (DP) and Scougall Bay (ScB; S7B, S7D and S7F Fig). The assignment proportion from the DAPC analysis drastically decreased in both libraries from 0.7324 using the NoPops population map and 0.7254 with LHDL, to 0.5282 with Pops in library A and from 0.7887 with NoPops and 0.8028 with LHDL to 0.6408 in the Pops population map in library B (S6 Fig). Fine scale population subdivision was detected with both libraries with Search Bay (SB), North Island (NI) and North Point (NP) differentiated in library A and Search Bay (SB) and North Point (NP) in library B in all three population maps (S8 Fig). In contrast, maximum likelihood analyses using ADMIXTURE was only able to resolve the Dore Lake samples using the NoPops population map in library A with an average ancestry fraction of 0.9304 (SD = 0.0684), while the LHDL and Pops population maps resulted in average ancestry fractions of 0.8412 (SD = 0.1857) and 0.9747 (SD = 0.0988), respectively (S9A, S9C and S9E Fig). Comparatively, with library B we could resolve strong population differentiation using all population maps with average ancestry fractions of 0.9755 (SD = 0.0261), 0.9501 (SD = 0.0751) and 0.9256 (SD = 0.1039) using the NoPops, LHDL and Pops population maps, respectively (S9B, S9D and S9F Fig). Within Lake Huron, no distinct population subdivision was detected using library A, while Search Bay (SB) and Hammond Bay (HB) showed differentiation using the NoPops and LHDL population maps in library B (S10 Fig). Increasing the number of populations required to contain the locus (-p) using the Pops population map resulted in a higher genotyping rate and less clustering based on sample site based on missing data in the IBM plot (S11 and S12 Fig; S1 File). Clustering based on missing data was apparent in the 9mer library until p6 (S12 Fig). Similarly, higher p values did not result in increased resolution in either the DAPC or ADMIXTURE analyses, with optimal resolution occurring using intermediate p values (S13 and S14 Figs).

The minimum percentage of individuals required to contain a locus within a population (-r) was tested from 0.1–0.8, corresponding to 10–80%, referred to here as r10-r80. The number of polymorphic loci generated after the *populations* module was 107.4X and 16.7X higher using r10 compared to r80 in library A and B, respectively (S15A Fig). The proportion of loci genotyped per individual was higher using library B at all -r values compared to library A with 65.06% (SD = 19.60%) and 17.90% (SD = 6.58%), respectively (S15B Fig). The GENODIVE analysis was able to resolve differentiation between the two lakes in both sequencing libraries regardless of the -r value (S16 Fig). The Dore Lake samples in library A had decreasing average F_ST_ values of 0.091 (SD = 0.004), 0.078 (SD = 0.003), 0.056 (SD = 0.002) and 0.038 (SD = 0.002) for r10, r30, r50 and r70, respectively (S16A, S16C, S16E and S16G Fig). Conversely, the F_ST_ values in library B were consistently high with average values of 0.124 (SD = 0.006), 0.131 (SD = 0.004), 0.131 (SD = 0.005) and 0.127 (SD = 0.003) for r10, r30, r50 and r70, respectively (S16B, S16D, S16F and S16H Fig). In all -r values in library B Search Bay (SB) showed slight differentiation within the Lake Huron samples, while this was not detectable in library A until very slight differentiation at r70 (S17 Fig). Both sequencing libraries were able to fully resolve Dore Lake in the DAPC analysis at all r values (S18 Fig). In library A, North Point (NP), North Island (NI) and Search Bay (SB) showed slight differentiation within Lake Huron in the r10 and r30 libraries and slightly in r50, while Search Bay (SB) and North Point (NP) showed consistent strong differentiation in library B (S19 Fig). Bayesian analyses using ADMIXTURE were clearly able to resolve Dore Lake in library A in the r10 and r30 libraries, while this signal was lost in the r50 and r70 datasets (S20A, S20C, S20E and S20G Fig). In contrast, library B was able to resolve strong differentiation at all -r values (S20B, S20D, S20F and S20H Fig). Library A was unable to resolve fine population differentiation using ADMIXTURE in Lake Huron with all -r values, m values and population maps, while Hammond Bay (HB) and Search Bay (SB) showed differentiation in library B (S21 Fig).

### Population differentiation

As a result of the higher number of loci and increased sequencing depth, library B was used to determine actual population differentiation. The m3 dataset was used as it resulted in the same level of differentiation in all three analyses, while retaining the most polymorphic loci (S4 Fig). The NoPops population map was used to maximize the genotyping rate (S4B Fig) and because there was very little population differentiation by missing data in the IBM analysis (Fig 3). Finally, an -r value of 30% was used in the following analyses as it resulted in a moderate level of loci with relatively low levels of missing data (S14 Fig).

**Fig 8:**
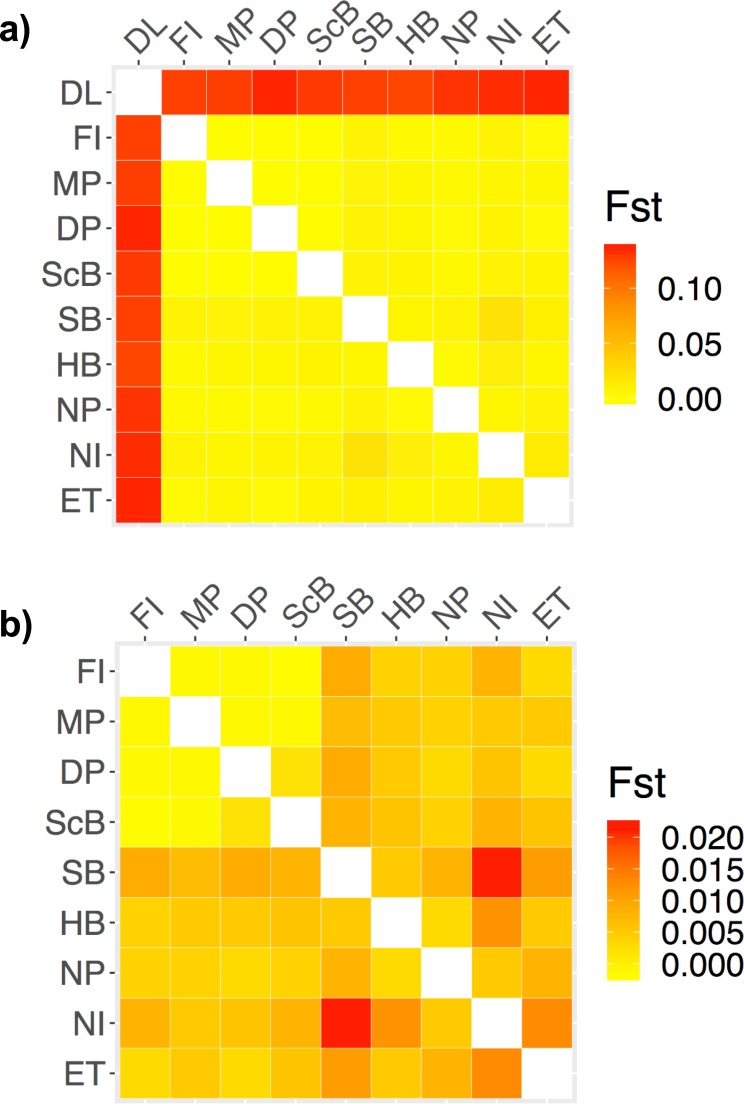
**Heatmap representing the pairwise fixation indices (F**_**ST**_**) estimated using GENODIVE across all sample sites (a) and only Lake Huron sites (b) in library B.** Larger F_ST_ values represent larger population differentiation. Site abbreviations can be found in Table 1.

**Fig 9:**
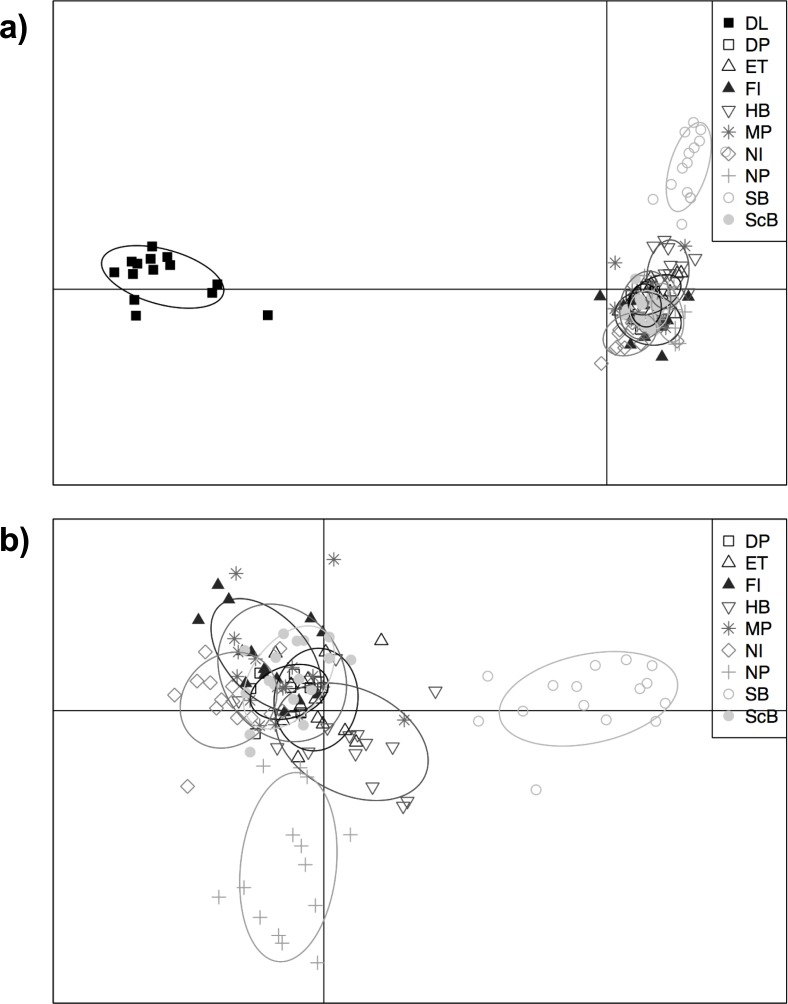
**Discriminant analysis of principle components (DAPC) of all sample sites (a) and only Lake Huron sites (b) using library B.** The DAPC analysis was run with 46 and 40 principle components (N/3) for the analysis with all sample sites and Lake Huron sites, respectively. Distinct ellipses indicate population differentiation. Site abbreviations can be found in Table 1.

**Fig 10:**
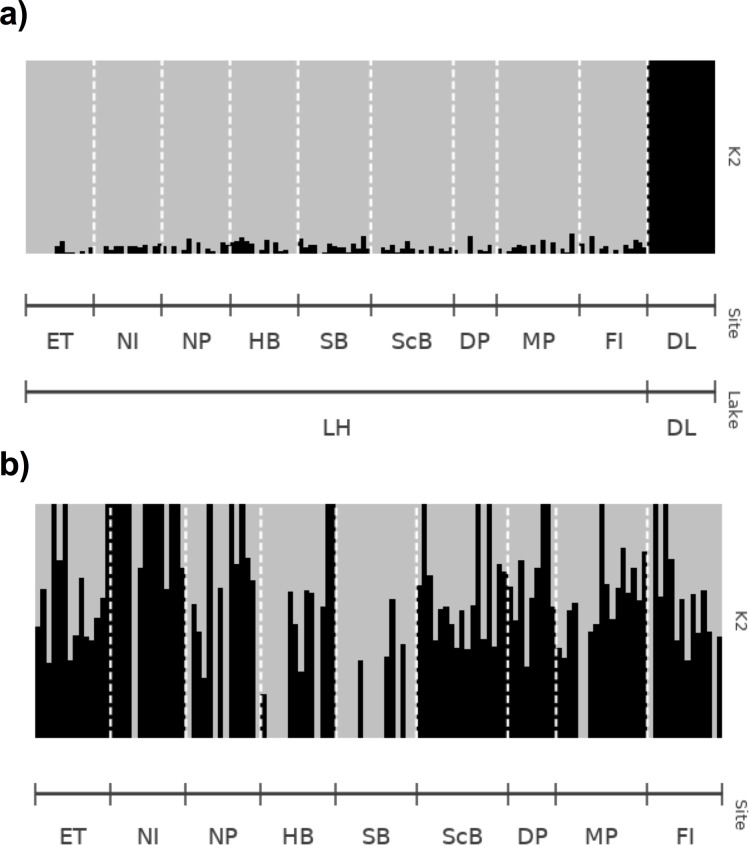
**ADMIXTURE analysis of all sample sites (a) and sites in Lake Huron (b) in library B.** K2 was used as it had the lowest cross-validation value post-hoc. Each line represents an individual from the corresponding sample site.

We used GENODIVE to determine pairwise F_ST_ values between lakes and each sample site (Fig 8). Dore Lake and Lake Huron showed significant differentiation with an F_ST_ value of 0.118 (P ≤ 0.001). Each sample site was then run in a pairwise F_ST_ analysis (Table 4; Fig 8A). Within the samples from Lake Huron, the average F_ST_ value was 0.038 (SD = 0.138; Table 3; Fig 8B). Within Lake Huron, Search Bay (SB) and North Island (NI) showed differentiation from the other sample sites as well as the sites in eastern Lake Huron, Fishing Island (FI), McRae Point (MP), Douglas Point (DP) and Scougall Bay (ScB; Fig 8B). DAPC was run using 46 and 40 principle components (N/3) in the analysis with all sample sites and the Lake Huron sites, respectively (Fig 9). The assignment proportion when analyzing all of the sample sites was 0.7887 (Fig 9A) and 0.7344 for the sample sites in Lake Huron (Fig 9B). Dore Lake showed strong differentiation, while North Point (NP) and Search Bay (SB) showed differentiation within Lake Huron (Fig 9). ADMIXTURE was run using all of the sample sites (Fig 10A) and only the individuals from Lake Huron (Fig 10B). In both cases, K = 2 resulted in the lowest value using the cross-validation approach *post hoc*. The analysis resulted in an average ancestry proportion of 0.9755 (SD = 0.0261) in the Dore Lake analysis (Fig 10A) and 0.7773 (SD = 0.1919) when analysing only Lake Huron, where Search Bay (SB) and Hammond Bay (HB) showed potential differentiation (Fig 10B).

**Table 4:** Pairwise F_ST_ estimates from GENODIVE using library B. F_ST_ estimates are above the diagonal and corresponding p-values are below for each sample site. The bolded numbers represent significant F_ST_ values (P ≤ 0.05). The minimum sequencing depth (-m) was set to 3 and minimum percentage of individuals required to contain the locus (-r) was 30% using the NoPops population map.

	ET	NI	NP	HB	SB	ScB	DP	MP	FI	DL
ET	--	**0.013**	**0.008**	**0.005**	**0.011**	**0.006**	0.003	**0.005**	0.003	**0.136**
NI	0	--	**0.005**	**0.012**	**0.022**	**0.008**	**0.006**	**0.005**	**0.008**	**0.135**
NP	0.001	0.009	--	0.003	**0.008**	**0.004**	0.003	**0.004**	**0.004**	**0.132**
HB	0.035	0	0.063	--	**0.005**	**0.006**	**0.005**	**0.005**	**0.004**	**0.126**
SB	0	0	0	0.004	--	**0.008**	**0.009**	**0.007**	**0.009**	**0.128**
ScB	0.004	0	0.014	0.001	0	--	0.002	-0.001	-0.002	**0.13**
DP	0.236	0.021	0.142	0.041	0	0.155	--	-0.001	-0.001	**0.136**
MP	0.035	0.01	0.027	0.01	0	0.783	0.7	--	-0.001	**0.129**
FI	0.118	0.001	0.033	0.04	0	0.797	0.699	0.747	--	**0.128**
DL	0	0	0	0	0	0	0	0	0	--

## Discussion

Library content and sequencing effort have a profound influence on the quality and potential power of inference of SNP datasets generated via RRLs. Technical aspects of study design can therefore impact downstream population structure analysis, especially when assessing fine scale population subdivision. Overall, we found that both libraries were able to resolve large scale population subdivision, but when examining fine scale differentiation library B produced more consistent and reliable results. Library A was able to differentiate strong population differentiation between Dore Lake and Lake Huron with low stringency on bioinformatic parameters, but increasing the stringency resulted in fewer loci leading to decreased resolution. This trend was clear in library A where large scale population subdivision between lakes was detected using low-stringency bioinformatics parameters, both -m and -r, and the signal was not lost until high stringencies. Compared to published population structure studies, the loss of signal we observed occurred at much higher -m values (m15 or m20) and within the range of commonly used -r values (r70), where common cut-offs range from m3 –m5 and r35 –r75, respectively [13,14,16,18,19,29,33,83–85].

All three population analyses resulted in greater differentiation between samples from Dore Lake and Lake Huron in library B for all parameters tested, with an average F_ST_ value consistently double, and both ancestry coefficients and assignment proportions ~10% higher than for library A. In contrast, when trying to detect fine scale population subdivision within Lake Huron, library A was either unable to detect subdivision, or yielded inconsistent results across analyses and with changing parameters; library B was much more consistent. Although the average sequencing depth in both libraries was relatively similar in the m3 datasets, the increased depth in library B resulted in higher genotype accuracy with more loci, generating a higher quality dataset [3,27,28,35,86]. Fountain et al. [37] investigated sequencing depth in parentage analyses and found that low-depth sequencing libraries resulted in higher genotyping error rates and increasing bioinformatic thresholds lead to large losses of loci. Here, we saw that the small decrease in depth of sequencing in library A lead to large losses of loci when increasing the sequencing depth parameter and also lower genotyping rates, which resulted in a loss of detection power for fine-scale population subdivision. These results indicate that when investigating population differentiation, it is not possible to rescue poor sequencing effort by increasing the stringency of bioinformatic parameters. In general, we found that detection of large scale differentiation was more robust to the effects of sequencing library quality and varying bioinformatic parameters, while the impacts on fine scale subdivision was more profound and generated inconsistent results.

Population designation (popmap) at the end of the bioinformatic pipeline influenced the amount of missing data introduced into the final dataset and also had significant impacts on the ability to differentiate strong subdivision. Similar to increasing the stringency on other parameters, changing the population specifications of individuals within the population map in the *populations* module lead to a loss of strong population differentiation and inconsistent results in the fine scale analyses in library A. In contrast, library B was always able to detect strong population subdivision and produced consistent results in the fine scale analysis. This difference is likely a result of the decrease in genotyping rate in library A combined with an overall decrease in the number of loci in the final dataset. Even though changing the population map to lake or site-specific labels lead to an increased number of loci in both libraries, these loci had large amounts of missing data that resulted in clustering based on similarities in the lack of a genotype, rather than true population structure. This is visible in the IBM plot and also DAPC where the sample sites become more defined by changing the population maps (Figs 7 and S3). This trend is also present when only one population was required to contain the locus using a population map with sample site designations in library A, where we see a large number of loci (>20,000) but the low genotyping rate drowns out the signal for strong differentiation in the maximum likelihood analysis (S13 Fig).

The impacts of missing data have been investigated in phylogenomic studies, which showed increased resolution with loosened thresholds and correspondingly reduced stringency [21,39,50–54]. However, studies investigating the influence of missing data have been limited to the impacts of various parameters on the final dataset [48,49], genotyping error rates [37] and different bioinformatic pipelines [47], with few studies investigating downstream population analyses. We found that the amount and distribution of missing data were influenced by multiple bioinformatics parameters acting together in ways that might not seem immediately obvious. Use of more specific population maps and low values for locus presence parameters (-p and -r) will result in a larger dataset with more loci. However, these same settings also resulted in more missing data and a decreased genotyping rate, which generated artifacts affecting the interpretation of downstream clustering and differentiation analyses. For example, missing data ultimately caused the loss of differentiation between Dore Lake and Lake Huron in our datasets based on library A. Importantly, in our study we held the number of populations requiring a locus (-p) at 1, and the percentage of individuals within that population (-r) at 30% as we made the applied population maps more specific. These settings create a very biased scenario that is unlikely to be used because they allow retention of all population-specific SNPs, thereby generating more and more discordance among datasets as the population map is adjusted. However, the analyses we present highlight just how important it is to understand the potential influence selection of these parameters can have on population structure studies. Common practice is to select arbitrary -p and -r values (e.g., often 50 to 70% for both parameters), which may still generate potential issues with missing data and biased population structure assessments based on the same concept we have illustrated here. The application of user-defined population maps and the setting for the p- and r-parameters need to be approached with care to strike the right balance between retaining more loci and generating confounding levels of missing data.

Increasing the stringency of various bioinformatic parameters reduces the number of loci present in the final dataset, which impacts population differentiation analyses. Specifically, increasing the sequencing depth threshold (-m) and proportion of individuals genotyped (-r) removes loci early in the pipeline with too few reads per individual. The remaining loci have higher average sequencing depth and genotyping rates, but many fewer loci remain with each iterative increase. The incorporation of high parameters early in the pipeline limits the final dataset of library A to loci with high sequencing depth, which are likely repetitive regions in the genome that are potentially less informative for examining population differentiation. The whole genome duplication event in the teleost genome exacerbates this issue in salmonids, creating many paralogous regions throughout the genome [25,87–90]. Further, setting the sequencing threshold parameter (-m) too high in a low sequencing depth library can lead to allelic dropout and incorrect genotyping calls [26]. Allelic dropout, where only one allele is sequenced within an individual, is an inherent issue in RRL studies and leads to overestimation of population differentiation parameters such as F_ST_ and H_exp_ [3,44,49]. In this study we found that fewer loci with high sequencing depth and genotyping rates were unable to resolve strong population subdivision in library A, even with less missing data present in the final dataset. Conversely, the higher initial sequencing depth of library B allowed for more loci to remain with increased stringency, resulting in no loss of inference. The differences between libraries indicates that the main factor influencing the ability to detect population subdivision is the sequencing effort at the front end of a project, as this impacts the amount missing data and total loci in the final dataset. We found that library B with more loci sequenced at a higher depth was more robust to increasing stringency of the parameters, while library A lost the power of inference as a result of too few loci.

The different population differentiation analysis programs had varying sensitivities to missing data and other changes resulting from changing the bioinformatic parameters. Analyses using fixation indices and maximum likelihood approaches were unable to resolve strong population differentiation with increasing stringency of bioinformatic parameters in library A. In contrast, the ordination analysis using DAPC was always able to resolve between lake subdivision in library A. Similarly, Jombart et al. [79] found that DAPC was more consistent than Bayesian analyses at characterizing population differentiation. This difference in the power of inference in population subdivision may be a result of the removal of loci that are out of HWE, which is not an assumption for the DAPC analysis, unlike ADMIXTURE and GENODIVE. Loci out of HWE are potentially produced by selection occurring within a population, unlike outlier loci which results from selection pressures across multiple populations and generations [91]. The removal of loci that deviate from HWE could result in the loss of important biological information [92]. Further, each analysis program treats missing data differently, with DAPC inferring genotypes based on the global average [79] and ADMIXTURE ignoring missing data [93], while GENODIVE employs pairwise comparisons where only genotypes present in the two populations are evaluated [78]. Overall, we found that each analysis has advantages and pitfalls that may be influenced by the removal of loci and missing data, and we recommend that biological inference be evaluated based on multiple approaches.

### Population differentiation

Strong population differentiation was detected between Lake Huron and Dore Lake in all three analyses using our best SNP dataset. These populations were physically separated several thousand years ago following dispersal after the Wisconsin glaciation, [60,94–96]. Within Lake Huron, all three analyses showed relatively strong differentiation of Search Bay from the rest of the sites, while North Point and Hammond Bay showed slight differentiation in the ordination and Bayesian approaches. This result is similar to previous microsatellite studies that found spawning populations near Lake Michigan were slightly genetically differentiated from the rest of the main basin in Lake Huron [69,70]. Further, Ebener et al. [97] performed a tag-recapture study that found evidence of movement of lake whitefish between Lake Huron and Lake Michigan, which was confirmed by a microsatellite study from Stott et al. [69] who found evidence of gene flow between the two lakes. Previous work in our lab using δC^13^ and δN^15^ stable isotopes also found that the individuals from the Search Bay and Hammond Bay spawning aggregations showed differentiation from the other western main basin samples [66]. However, in contrast to the results obtained here, these samples also clustered with the sites on the eastern main basin, including Fishing Islands, Scougall Bay, Douglas Point and McRae Point [66].

### Conclusion

In this study we show the importance of investing in an appropriate DNA library and sequencing effort at the beginning of a study to ensure the best possible population structure inference. Specifically, we recommend investing in extra sequencing depth at a moderate amount of loci to address the expected level of population differentiation, with more sequencing effort required for fine scale studies. Despite the added cost per locus sequenced, it is better to err on the side of excess sequencing effort (number of loci and sequencing depth) and trim datasets, rather than be forced to use smaller or suboptimal datasets in analyses. The required sequencing depth is a consistent matter of debate in next-generation DNA sequencing studies. Unfortunately, there is no one-size-fits-all formula for this critical sequencing target. Generally, more loci and higher sequencing depth is better for population structure studies to maintain genotyping accuracy and resolution power. Based on loci lost during quality control and bioinformatics filtering, a good general target at the outset of a study is to aim for 8-10X more loci than required after filtering [14,20,98,99]. Here, library B generated ~8.5X more loci than was present in the final dataset at 12X sequencing depth. Following sequencing, we show the importance of testing for optimal bioinformatic values and avoiding the use of overly-stringent bioinformatic parameters, which may excessively reduce datasets, and are not capable of rescuing poor sequencing efforts. Similar to previous studies, we found that correct filtering of data has a large impact on data interpretation and the quality of the final dataset, especially with low sequencing effort [49,92]. The quality of the sequence data and the stringency of the bioinformatic parameters has a drastic influence on the number of informative loci in the final dataset and the downstream population analyses and therefore should be examined to allow for confidence in biological inference.

## Supporting information

S1 File Investigating the impacts of the minimum number of populations required to contain a locus (-p) with a predefined population map (Pops). (DOCX)

S1 Fig **Heatmap representing the pairwise fixation indices (F**_**ST**_**) estimated using GENODIVE across sample sites found in Lake Huron with 5 sequencing depth thresholds (m) in the *ustacks* module of STACKS with m3 (a, b), m6 (c, d), m10 (e, f), m15 (g, h) and m20 (i, j) in library A (a, c, e, g, i) and library B (b, d, f, h, j)**. No population designations were used (NoPops)in the *populations* module and a and minimum of 30% of individuals were required to contain the locus (-r). Larger F_ST_ values represent larger population differentiation. Sites sampled in Lake Huron were Fishing Islands (FI), McRae Point (MP), Douglas Point (DP), Scougall Bay (ScB), Search Bay (SB), Hammond Bay (HB), North Point (NP), North Island (NI) and East Tawas (ET).
(EPS)

S2 Fig **Discriminant analysis of principle components (DAPC) of Lake Huron sample sites with increasing sequencing depth (m) in the *ustacks* module with m3 (a, b), m6 (c, d), m10 (e, f), m15 (g, h) and m20 (i, j) in library A (a, c, e, g, i) and library B (b, d, f, h, j).** The DAPC analysis was run with 40 principle components (N/3). No population designations were used (NoPops)in the *populations* module and a and minimum of 30% of individuals were required to contain the locus (-r). Distinct ellipses indicate population differentiation. Sites sampled in Lake Huron were Fishing Islands (FI), McRae Point (MP), Douglas Point (DP), Scougall Bay (ScB), Search Bay (SB), Hammond Bay (HB), North Point (NP), North Island (NI) and East Tawas (ET).
(EPS)

S3 Fig **ADMIXTURE analysis of the Lake Huron sample sites with increasing sequencing depth (m) in the *ustacks* module with m3 (a, b), m6 (c, d), m10 (e, f), m15 (g, h) and m20 (i, j) in library A (a, c, e, g, i) and library B (b, d, f, h, j).** K2 was used as it had the lowest cross-validation value post-hoc. Each line represents an individual from the corresponding sample site. Sites sampled in Lake Huron were Fishing Islands (FI), McRae Point (MP), Douglas Point (DP), Scougall Bay (ScB), Search Bay (SB), Hammond Bay (HB), North Point (NP), North Island (NI) and East Tawas (ET).
(EPS)

S4 Fig **The proportion of loci genotyped in each individual in library (a) A and (b) B with increasing sequencing depth values (-m) in *ustacks*.** Data were generated using three different population maps in the *populations* module of STACKS, no specified populations (NoPops), Lake Huron and Dore Lake samples (LHDL) or sample sites (Pops). The box represents the interquartile range of the data, the line in the middle is the median, and the lines above and below represent the maximum and minimum, respectively.
(TIFF)

S5 Fig **Heatmap representing the pairwise fixation indices (F**_**ST**_**) estimated using GENODIVE across all sample sites with 3 different population maps in the *populations* module: (1) no population differentiation (NoPops; a, b), (2) Dore Lake and Lake Huron designations (LHDL; c, d) and (3) sample site designations (Pops; e, f) in both library A (a, c, e) and B (b, d, f).** The minimum sequencing depth (-m) was set at 3 and minimum percentage of individuals required to contain the locus (-r) was 30%. Larger F_ST_ values represent larger population differentiation. Sites were sampled in Dore Lake (DL) and nine locations in Lake Huron: Fishing Islands (FI), McRae Point (MP), Douglas Point (DP), Scougall Bay (ScB), Search Bay (SB), Hammond Bay (HB), North Point (NP), North Island (NI) and East Tawas (ET).
(EPS)

S6 Fig **Discriminant analysis of principle components (DAPC) of all sample sites with 3 different population maps in the *populations* module: (1) no population differentiation (NoPops; a, b), (2) Dore Lake and Lake Huron designations (LHDL; c, d) and (3) sample site designations (Pops; e, f) in both library A (a, c, e) and B (b, d, f).** The minimum sequencing depth (-m) was set at 3 and minimum percentage of individuals required to contain the locus (-r) was 30%. The DAPC analysis was run with 46 principle components (N/3) for the analysis. Distinct ellipses indicate population differentiation. Sites were sampled in Dore Lake (DL) and nine locations in Lake Huron: Fishing Islands (FI), McRae Point (MP), Douglas Point (DP), Scougall Bay (ScB), Search Bay (SB), Hammond Bay (HB), North Point (NP), North Island (NI) and East Tawas (ET).
(EPS)

S7 Fig **Heatmap representing the pairwise fixation indices (F**_**ST**_**) estimated using GENODIVE across Lake Huron sample sites with 3 different population maps in the *populations* module: (1) no population differentiation (NoPops; a, b), (2) Dore Lake and Lake Huron designations (LHDL; c, d) and (3) sample site designations (Pops; e, f) in both library A (a, c, e) and B (b, d, f).** The minimum sequencing depth (-m) was set at 3 and minimum percentage of individuals required to contain the locus (-r) was 30%. Larger F_ST_ values represent larger population differentiation. Sites sampled in Lake Huron were Fishing Islands (FI), McRae Point (MP), Douglas Point (DP), Scougall Bay (ScB), Search Bay (SB), Hammond Bay (HB), North Point (NP), North Island (NI) and East Tawas (ET).
(EPS)

S8 Fig **Discriminant analysis of principle components (DAPC) of Lake Huron sites with 3 different population maps in the *populations* module: (1) no population differentiation (NoPops; a, b), (2) Dore Lake and Lake Huron designations (LHDL; c, d) and (3) sample site designations (Pops; e, f) in both library A (a, c, e) and B (b, d, f).** The minimum sequencing depth (-m) was set at 3 and minimum percentage of individuals required to contain the locus (-r) was 30%. The DAPC analysis was run with 46 principle components (N/3) for the analysis. Distinct ellipses indicate population differentiation. Sites sampled in Lake Huron were Fishing Islands (FI), McRae Point (MP), Douglas Point (DP), Scougall Bay (ScB), Search Bay (SB), Hammond Bay (HB), North Point (NP), North Island (NI) and East Tawas (ET).
(EPS)

S9 Fig **ADMIXTURE analysis of all sample sites with 3 different population maps in the *populations* module: (1) no population differentiation (NoPops; a, b), (2) Dore Lake and Lake Huron designations (LHDL; c, d) and (3) sample site designations (Pops; e, f) in both library A (a, c, e) and B (b, d, f).** The minimum sequencing depth (-m) was set at 3 and minimum percentage of individuals required to contain the locus (-r) was 30%. K2 was used as it had the lowest cross-validation value post-hoc. Each line represents an individual from the corresponding sample site. Sites were sampled in Dore Lake (DL) and nine locations in Lake Huron: Fishing Islands (FI), McRae Point (MP), Douglas Point (DP), Scougall Bay (ScB), Search Bay (SB), Hammond Bay (HB), North Point (NP), North Island (NI) and East Tawas (ET).
(TIFF)

S10 Fig **ADMIXTURE analysis of the Lake Huron sample sites with 3 different population maps in the *populations* module: (1) no population differentiation (NoPops; a, b), (2) Dore Lake and Lake Huron designations (LHDL; c, d) and (3) sample site designations (Pops; e, f) in both library A (a, c, e) and B (b, d, f).** The minimum sequencing depth (-m) was set at 3 and minimum percentage of individuals required to contain the locus (-r) was 30%. K2 was used as it had the lowest cross-validation value post-hoc. Each line represents an individual from the corresponding sample site. Sites sampled in Lake Huron were Fishing Islands (FI), McRae Point (MP), Douglas Point (DP), Scougall Bay (ScB), Search Bay (SB), Hammond Bay (HB), North Point (NP), North Island (NI) and East Tawas (ET).
(EPS)

S11 Fig **The number of polymorphic loci (a) and the proportion of loci genotyped (b) with increasing minimum numbers of populations required to contain the locus.** The box represents the interquartile range of the data, the line in the middle is the median, and the lines above and below represent the maximum and minimum, respectively.
(EPS)

S12 Fig **Identity-by-missingness (IBM) plot generated using *grur* with increasing p values in library A using the Pops population map in the *population* module of STACKS: p1 (a), p2 (b), p3 (c), p4 (d), p5 (e), p6 (f), p7 (g), p8 (h), p9 (i), p10 (j)**. The minimum sequencing depth (-m) was set at 3 and minimum percentage of individuals required to contain the locus (-r) was 30%. The IBM analysis is conducted using a Principal Coordinates Analysis (PCoA) to determine clustering based on missing data. The x and y axes represent the first and second principal coordinate, respectively. Sites were sampled in Dore Lake (DL) and nine locations in Lake Huron: Fishing Islands (FI), McRae Point (MP), Douglas Point (DP), Scougall Bay (ScB), Search Bay (SB), Hammond Bay (HB), North Point (NP), North Island (NI) and East Tawas (ET).
(EPS)

S13 Fig **Discriminant analysis of principle components (DAPC) plots of lake whitefish from Dore Lake (DL) and Lake Huron with a minimum number of populations containing the locus of (a) 1, (b) 5 and (c) 10 in library A and (d) 1, (e) 5 and (f) 10 in library B**. The minimum sequencing depth (-m) was set at 3, the minimum percentage of individuals required to contain the locus (-r) was 30% and sample site designations (Pops) were used in the population map. Non-overlapping ellipses indicate population differentiation. Sites were sampled in Dore Lake (DL) and nine locations in Lake Huron: Fishing Islands (FI), McRae Point (MP), Douglas Point (DP), Scougall Bay (ScB), Search Bay (SB), Hammond Bay (HB), North Point (NP), North Island (NI) and East Tawas (ET).
(EPS)

S14 Fig **ADMIXTURE plots of lake whitefish from Dore Lake (DL) and Lake Huron (LH) using (a) library A (b) B with K = 2.** Each plot shows increasing numbers of populations required to contain the locus with p1, p5 and p10. The minimum sequencing depth (-m) was set at 3, the minimum percentage of individuals required to contain the locus (-r) was 30% and sample site designations (Pops) were used in the population map. Each bar represents a single individual with the colour corresponding to the ancestry fraction to each group. Sites were sampled in Dore Lake (DL) and nine locations in Lake Huron: Fishing Islands (FI), McRae Point (MP), Douglas Point (DP), Scougall Bay (ScB), Search Bay (SB), Hammond Bay (HB), North Point (NP), North Island (NI) and East Tawas (ET).
(TIFF)

S15 Fig **The number of polymorphic loci (a) and the proportion of genotyped loci (b) with increasing minimum percentage of individuals within a population required to have a locus in library A (black) and library B (gray).** The box represents the interquartile range of the data, the line in the middle is the median, and the lines above and below represent the maximum and minimum, respectively.
(EPS)

S16 Fig **Heatmap representing the pairwise fixation indices (F**_**ST**_**) estimated using GENODIVE across all sample sites increasing the minimum percentage of individuals required to contain a locus (r) in the *populations* module with r10 (a, b), r30 (c, d), r50 (e, f) and r70 (g, h) in both library A (a, c, e, g) and B (b, d, f, h).** The minimum sequencing depth (-m) was set at 3 and no sample site designations (NoPops) were used in the population map. Larger F_ST_ values represent larger population differentiation. Sites were sampled in Dore Lake (DL) and nine locations in Lake Huron: Fishing Islands (FI), McRae Point (MP), Douglas Point (DP), Scougall Bay (ScB), Search Bay (SB), Hammond Bay (HB), North Point (NP), North Island (NI) and East Tawas (ET).
(EPS)

S17 Fig **Heatmap representing the pairwise fixation indices (F**_**ST**_**) estimated using GENODIVE across Lake Huron sample sites increasing the minimum percentage of individuals required to contain a locus (r) in the *populations* module with r10 (a, b), r30 (c, d), r50 (e, f) and r70 (g, h) in both library A (a, c, e, g) and B (b, d, f, h).** The minimum sequencing depth (-m) was set at 3 and no sample site designations (NoPops) were used in the population map. Larger F_ST_ values represent larger population differentiation. Sites sampled in Lake Huron were Fishing Islands (FI), McRae Point (MP), Douglas Point (DP), Scougall Bay (ScB), Search Bay (SB), Hammond Bay (HB), North Point (NP), North Island (NI) and East Tawas (ET).
(EPS)

S18 Fig **Discriminant analysis of principle components (DAPC) plots of lake whitefish from Dore Lake (DL) and Lake Huron increasing the minimum percentage of individuals required to contain a locus (r) in the *populations* module with r10 (a, b), r30 (c, d), r50 (e, f) and r70 (g, h) in both library A (a, c, e, g) and B (b, d, f, h).** The minimum sequencing depth (-m) was set at 3 and no sample site designations (NoPops) were used in the population map. Non-overlapping ellipses indicate population differentiation. Sites were sampled in Dore Lake (DL) and nine locations in Lake Huron: Fishing Islands (FI), McRae Point (MP), Douglas Point (DP), Scougall Bay (ScB), Search Bay (SB), Hammond Bay (HB), North Point (NP), North Island (NI) and East Tawas (ET).
(EPS)

S19 Fig **Discriminant analysis of principle components (DAPC) plots of lake whitefish from Lake Huron sample sites increasing the minimum percentage of individuals required to contain a locus (r) in the *populations* module with r10 (a, b), r30 (c, d), r50 (e, f) and r70 (g, h) in both library A (a, c, e, g) and B (b, d, f, h).** The minimum sequencing depth (-m) was set at 3 and no sample site designations (NoPops) were used in the population map. Non-overlapping ellipses indicate population differentiation. Sites sampled in Lake Huron were Fishing Islands (FI), McRae Point (MP), Douglas Point (DP), Scougall Bay (ScB), Search Bay (SB), Hammond Bay (HB), North Point (NP), North Island (NI) and East Tawas (ET).
(EPS)

S20 Fig **ADMIXTURE plots of lake whitefish from Dore Lake (DL) and Lake Huron (LH) increasing the minimum percentage of individuals required to contain a locus (r) in the *populations* module with r10 (a, b), r30 (c, d), r50 (e, f) and r70 (g, h) in both library A (a, c, e, g) and B (b, d, f, h).** K2 was used as it had the lowest cross-validation value post-hoc. The minimum sequencing depth (-m) was set at 3 and no sample site designations (NoPops) were used in the population map. Each bar represents a single individual with the colour corresponding to the ancestry fraction to each group. Sites were sampled in Dore Lake (DL) and nine locations in Lake Huron: Fishing Islands (FI), McRae Point (MP), Douglas Point (DP), Scougall Bay (ScB), Search Bay (SB), Hammond Bay (HB), North Point (NP), North Island (NI) and East Tawas (ET).
(TIFF)

S21 Fig **ADMIXTURE plots of lake whitefish from Lake Huron (LH) increasing the minimum percentage of individuals required to contain a locus (r) in the *populations* module with r10 (a, b), r30 (c, d), r50 (e, f) and r70 (g, h) in both library A (a, c, e, g) and B (b, d, f, h). K2 was used as it had the lowest cross-validation value post-hoc.** The minimum sequencing depth (-m) was set at 3 and no sample site designations (NoPops) were used in the population map. Each bar represents a single individual with the colour corresponding to the ancestry fraction to each group. Sites sampled in Lake Huron were Fishing Islands (FI), McRae Point (MP), Douglas Point (DP), Scougall Bay (ScB), Search Bay (SB), Hammond Bay (HB), North Point (NP), North Island (NI) and East Tawas (ET).
(EPS)
