# Supplementary material for: How “simple” methodological decisions affect interpretation of population structure based on reduced representation library DNA sequencing: A case study using the lake whitefish
Source: PLoS One. 2020 Jan 24;15(1):e0226608. doi: 10.1371/journal.pone.0226608 (PMC6980518; doi:10.1371/journal.pone.0226608)
Supplement: S1 File — (DOCX) [file pone.0226608.s001.docx]

**File S1**

The minimum number of populations (p) required to contain a locus was tested from p1 – p10 using the sample site designations in the Pops population map. Library B had 1.3X – 3.5X more polymorphic loci than library A at all p values (Fig. S11a). With increasing p values the number of polymorphic loci decreased resulting in 14.5X and 5.5X more loci in the p1 dataset compared to p10 for library A and B, respectively (Fig. S11a). Library B also had a higher proportion of genotyped loci with an average of 55.84% (SD = 15.59%) genotyped across all p values compared to 41.60% (SD = 16.16%) in library A (Fig. S11b). With increasing p values the genotyping rate per individual increased 4.0- and 3.0-fold from p1 to p10 in library A and B, respectively (Fig. S11b).

Increasing the number of populations required to contain the locus (p) with the Pops population map also resulted in varying outcomes for population differentiation analyses in both DAPC (Fig. S12) and ADMIXTURE (Fig. S13) for library A and B. DAPC was able to resolve Dore Lake and Lake Huron samples regardless of the sequencing primer or p value with assignment proportions of 0.5282 for p1, 0.7535 for p5 and 0.8028 for p10 in library A and 0.6408 for p1, 0.7887 for p5 and 0.8451 for p10 in library B (Fig. S12). Weak population differentiation was also detected with Search Bay (SB), North Island (NI) and North Point (NP) showing slight differentiation in p1 and p5 of library A, while Search Bay (SB) shows differentiation in library B at all p values (Fig. S12). ADMIXTURE was unable to resolve this strong population structure with p1 or p10 in library A but resulted in strong differentiation at all p values in library B (Fig. S13).
