# Supplementary figures and images for: How “simple” methodological decisions affect interpretation of population structure based on reduced representation library DNA sequencing: A case study using the lake whitefish
Source: PLoS One. 2020 Jan 24;15(1):e0226608. doi: 10.1371/journal.pone.0226608 (PMC6980518; doi:10.1371/journal.pone.0226608)

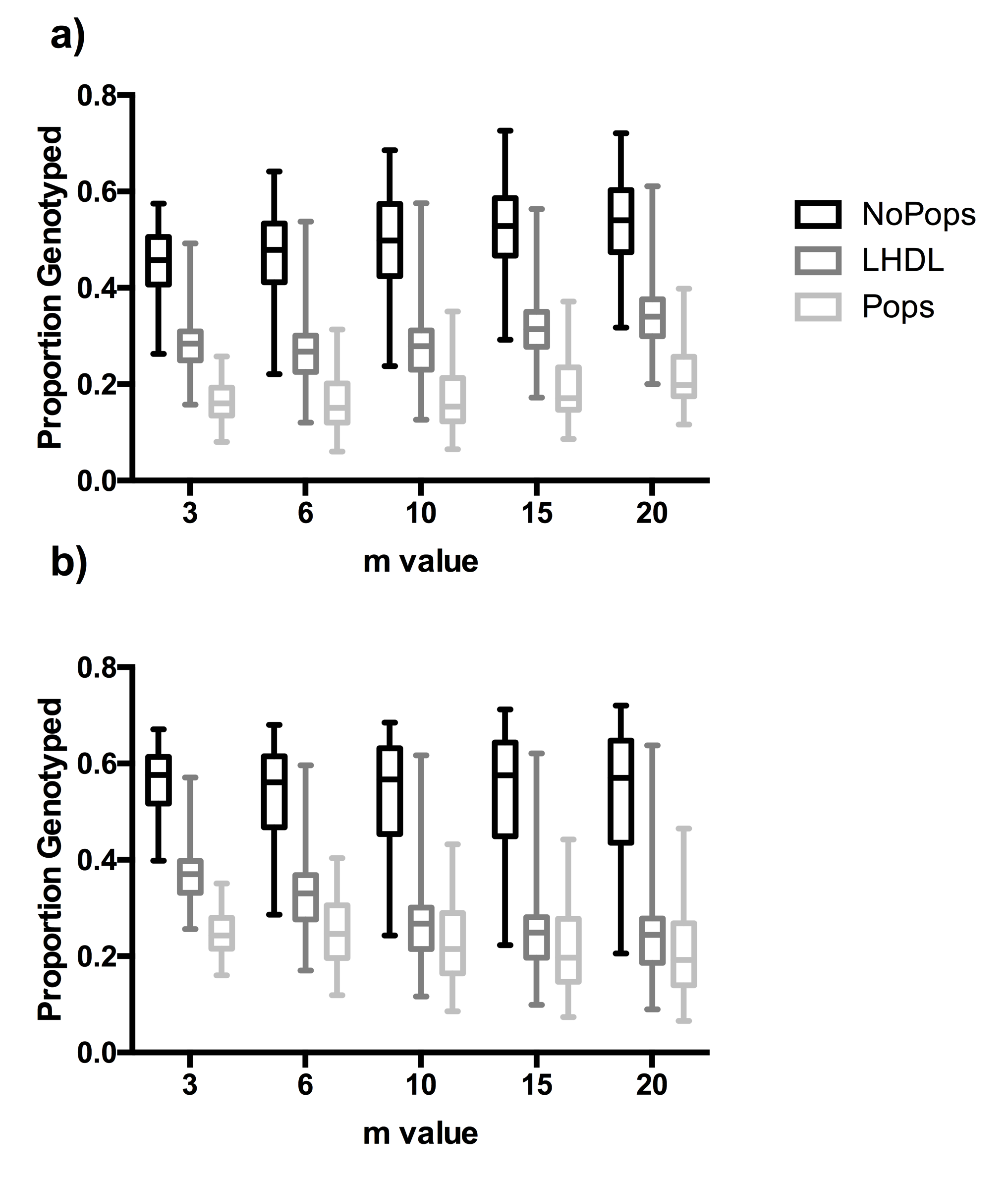

Supplement: S4 Fig — The proportion of loci genotyped in each individual in library (a) A and (b) B with increasing sequencing depth values (-m) in ustacks. Data were generated using three different population maps in the populations module of STACKS, no specified populations (NoPops), Lake Huron and Dore Lake samples (LHDL) or sample sites (Pops). The box represents the interquartile range of the data, the line in the middle is the median, and the lines above and below represent the maximum and minimum, respectively. (TIFF) [file pone.0226608.s005.tiff]

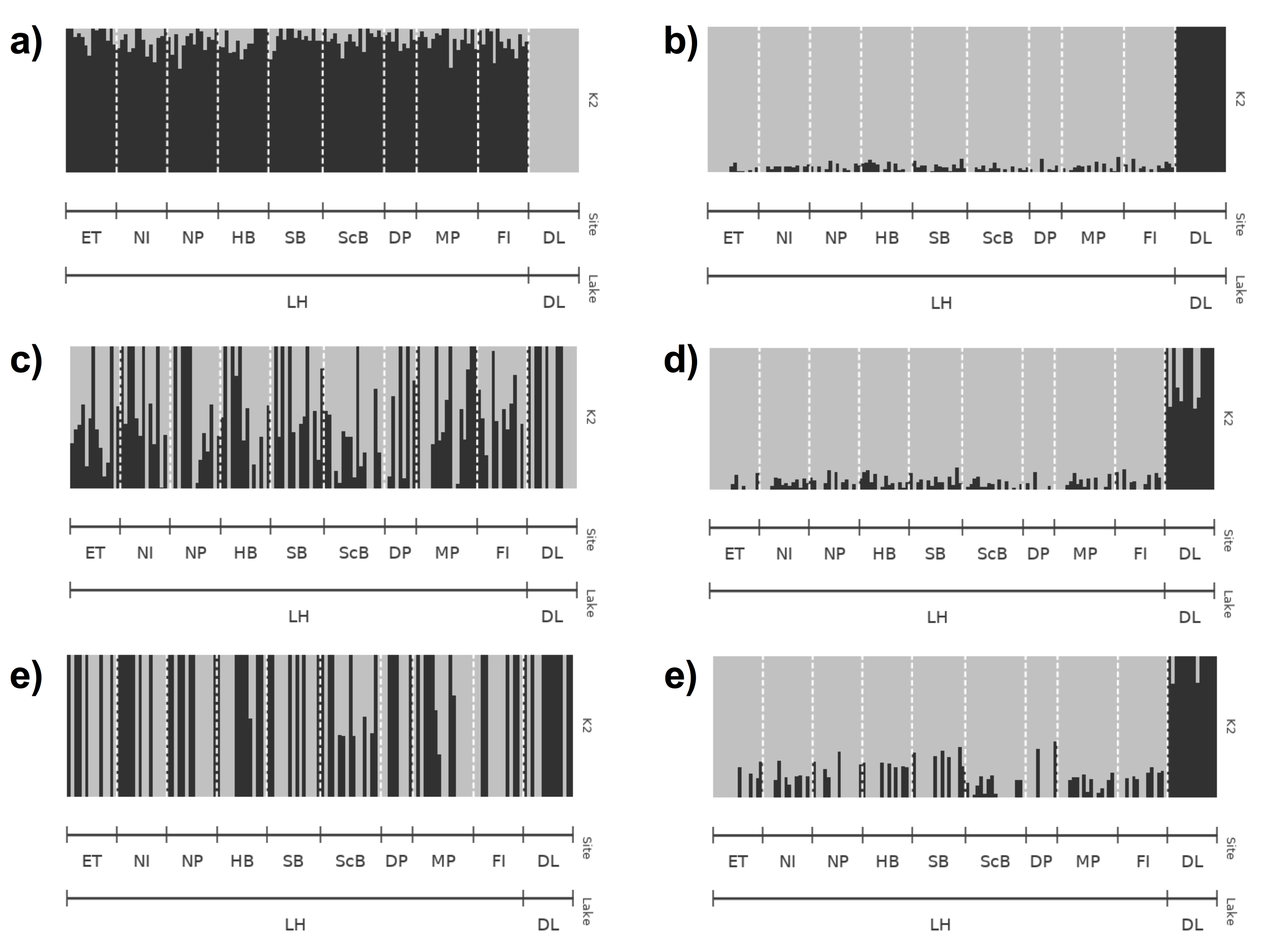

Supplement: S9 Fig — ADMIXTURE analysis of all sample sites with 3 different population maps in the populations module: (1) no population differentiation (NoPops; a, b), (2) Dore Lake and Lake Huron designations (LHDL; c, d) and (3) sample site designations (Pops; e, f) in both library A (a, c, e) and B (b, d, f). The minimum sequencing depth (-m) was set at 3 and minimum percentage of individuals required to contain the locus (-r) was 30%. K2 was used as it had the lowest cross-validation value post-hoc. Each line represents an individual from the corresponding sample site. Sites were sampled in Dore Lake (DL) and nine locations in Lake Huron: Fishing Islands (FI), McRae Point (MP), Douglas Point (DP), Scougall Bay (ScB), Search Bay (SB), Hammond Bay (HB), North Point (NP), North Island (NI) and East Tawas (ET). (TIFF) [file pone.0226608.s010.tiff]

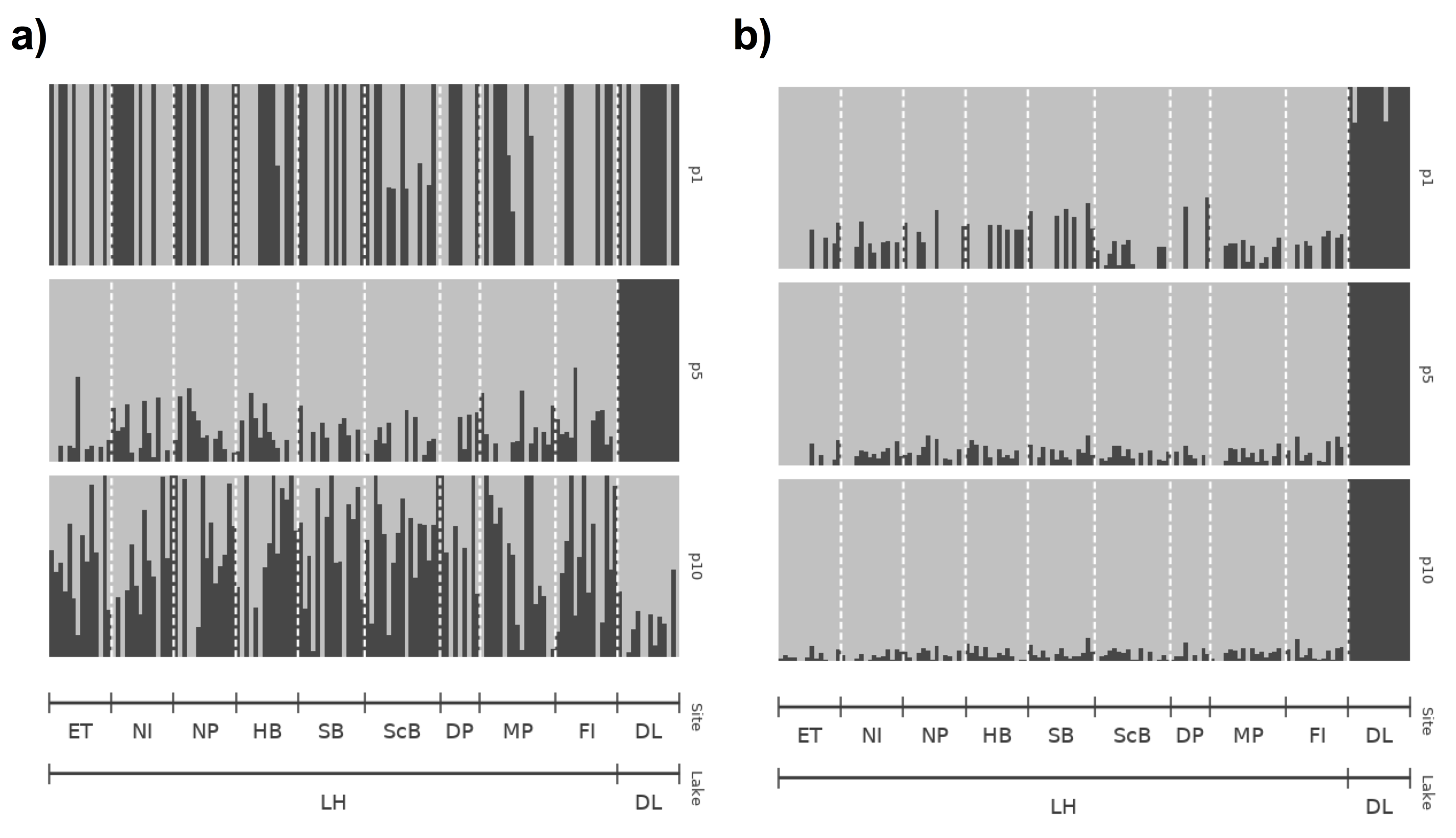

Supplement: S14 Fig — ADMIXTURE plots of lake whitefish from Dore Lake (DL) and Lake Huron (LH) using (a) library A (b) B with K = 2. Each plot shows increasing numbers of populations required to contain the locus with p1, p5 and p10. The minimum sequencing depth (-m) was set at 3, the minimum percentage of individuals required to contain the locus (-r) was 30% and sample site designations (Pops) were used in the population map. Each bar represents a single individual with the colour corresponding to the ancestry fraction to each group. Sites were sampled in Dore Lake (DL) and nine locations in Lake Huron: Fishing Islands (FI), McRae Point (MP), Douglas Point (DP), Scougall Bay (ScB), Search Bay (SB), Hammond Bay (HB), North Point (NP), North Island (NI) and East Tawas (ET). (TIFF) [file pone.0226608.s015.tiff]

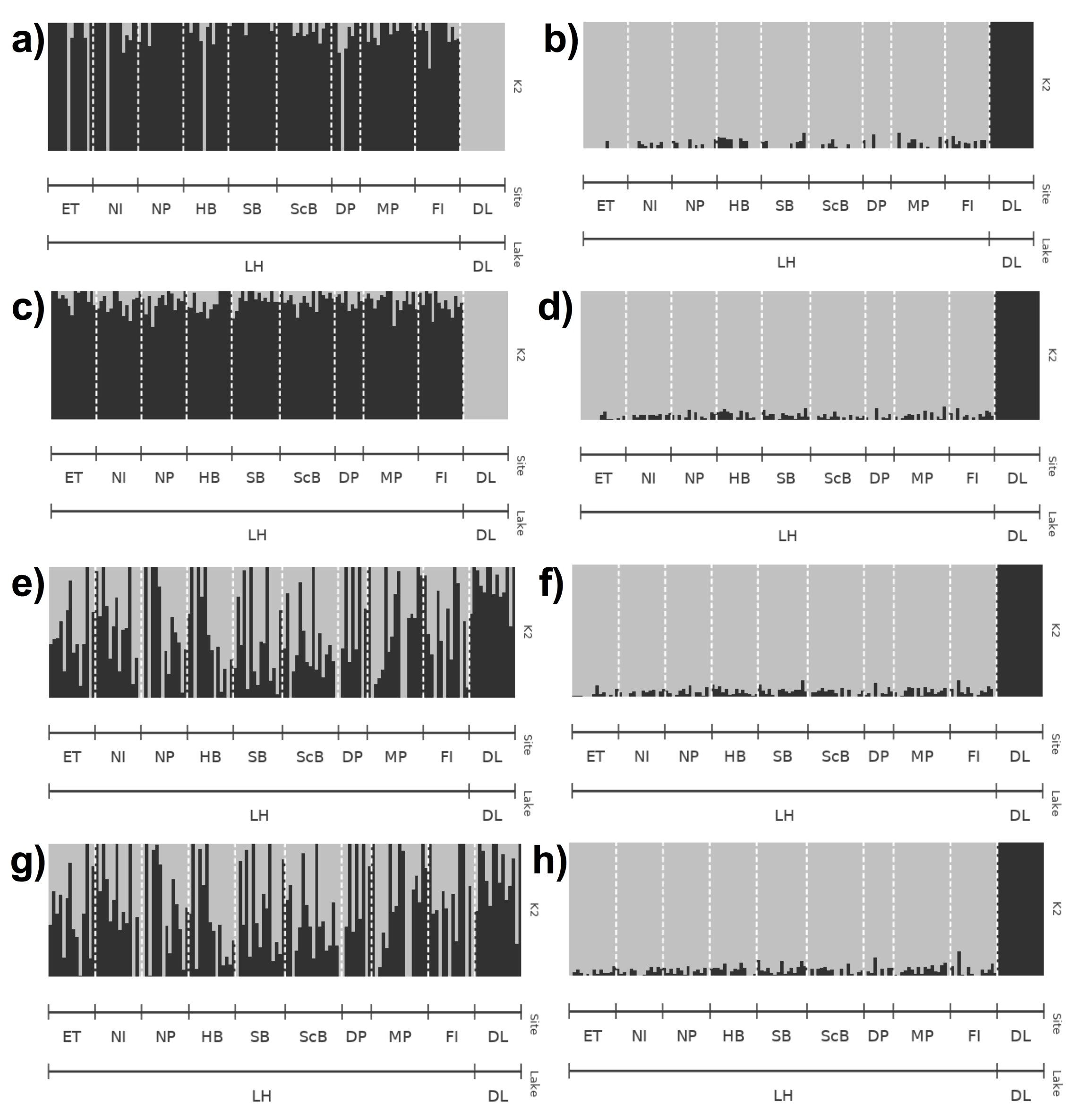

Supplement: S20 Fig — ADMIXTURE plots of lake whitefish from Dore Lake (DL) and Lake Huron (LH) increasing the minimum percentage of individuals required to contain a locus (r) in the populations module with r10 (a, b), r30 (c, d), r50 (e, f) and r70 (g, h) in both library A (a, c, e, g) and B (b, d, f, h). K2 was used as it had the lowest cross-validation value post-hoc. The minimum sequencing depth (-m) was set at 3 and no sample site designations (NoPops) were used in the population map. Each bar represents a single individual with the colour corresponding to the ancestry fraction to each group. Sites were sampled in Dore Lake (DL) and nine locations in Lake Huron: Fishing Islands (FI), McRae Point (MP), Douglas Point (DP), Scougall Bay (ScB), Search Bay (SB), Hammond Bay (HB), North Point (NP), North Island (NI) and East Tawas (ET). (TIFF) [file pone.0226608.s021.tiff]
